# Phosphoproteomics Sample Preparation Impacts Biological Interpretation of Phosphorylation Signaling Outcomes

**DOI:** 10.3390/cells10123407

**Published:** 2021-12-03

**Authors:** Bharath Sampadi, Leon H. F. Mullenders, Harry Vrieling

**Affiliations:** 1 Department of Human Genetics, Leiden University Medical Center, Einthovenweg 20, 2333 ZC Leiden, The Netherlands; leonmullenders@gmail.com; 2 Department of Genetics, Research Institute of Environmental Medicine (RIeM), Nagoya University, Nagoya 464-0805, Aichi, Japan

**Keywords:** high throughput phosphoproteomics, protein phosphorylation, signal transduction, DNA damage response, cell signaling, stress response, ionizing radiation, strong cation exchange chromatography

## Abstract

The influence of phosphoproteomics sample preparation methods on the biological interpretation of signaling outcome is unclear. Here, we demonstrate a strong bias in phosphorylation signaling targets uncovered by comparing the phosphoproteomes generated by two commonly used methods—strong cation exchange chromatography-based phosphoproteomics (SCXPhos) and single-run high-throughput phosphoproteomics (HighPhos). Phosphoproteomes of embryonic stem cells exposed to ionizing radiation (IR) profiled by both methods achieved equivalent coverage (around 20,000 phosphosites), whereas a combined dataset significantly increased the depth (>30,000 phosphosites). While both methods reproducibly quantified a subset of shared IR-responsive phosphosites that represent DNA damage and cell-cycle-related signaling events, most IR-responsive phosphoproteins (>82%) and phosphosites (>96%) were method-specific. Both methods uncovered unique insights into phospho-signaling mediated by single (SCXPhos) versus double/multi-site (HighPhos) phosphorylation events; particularly, each method identified a distinct set of previously unreported IR-responsive kinome/phosphatome (95% disparate) directly impacting the uncovered biology.

## 1. Introduction

Protein phosphorylation is a post-translational modification (PTM) that displays highly complex and dynamic properties with a dramatic low abundance compared to unmodified proteins. Hence, phosphorylated peptides need to be enriched from highly abundant non-phosphorylated peptides before successful identification by a mass spectrometer (MS) is possible without deep fractionation of the sample [1]. A crucial initial step in phosphoproteomics experiments is the sample preparation workflow involving lysate preparation, protein digestion, phosphopeptide enrichment and cleanup. While trypsin is highly specific and very effective in digesting proteins into peptides, it will not allow the detection of phosphopeptides that do not contain nearby R/K cleavage sites. In addition, phosphopeptides can be difficult to digest with trypsin since phosphorylation itself can inhibit the activity of the enzyme, mainly through the formation of salt bridges between R/K cleavage sites and phosphorylated serine or threonine residues [2]. The inhibition of phosphopeptide digestion reduces the number of phosphopeptides identified and hence negatively impacts their identification and quantification. Increasing the amount of trypsin employed only partially increases phosphopeptide cleavage depending on the phosphopeptide sequence [2]. Furthermore, most phosphoproteomics studies have only used trypsin to generate phosphoproteome data while applying the 1:100 trypsin-to-peptide ratio that is standardly used for global proteomics and may thus have failed to detect numerous biologically important phosphosites. In line with this notion, a recent large-scale phosphoproteomics study demonstrated that the usage of multiple proteases dramatically enhances phosphopeptide identification and the sequence coverage of phosphoproteins [3].

The enrichment of phosphopeptides can be performed by different methods that involve either a complex workflow achieving extensive coverage or a simple workflow achieving sufficient coverage of phosphoproteomes. The most common strategy for a complex workflow is to perform an initial peptide fractionation, typically using strong cation exchange (SCX) chromatography or, more recently, a powerful version of high-pH reversed-phase chromatography followed by the concatenation of fractions (bRP-fc). After this initial peptide fractionation, a second stage of purification is performed that involves the phosphopeptide enrichment of each peptide fraction using an immobilized metal affinity chromatography (IMAC) or a metal–oxide affinity chromatography (MOAC) step. Commonly used IMAC materials include Ga^3+^, Fe^3+^ and Ti^4+^ immobilized beads [4,5,6], whereas TiO_2_, ZrO_2_ and Al(OH)_3_ × H_2_O are the most commonly used MOAC materials [7]. While complex multi-step workflows enable extensive coverage of phosphoproteomes, they are resource-, time-, and labor-intensive. Experiments involving a complex multi-step workflow also often require specialized equipment and several processing steps that can affect the robustness and reproducibility of experiments.

Single-run phosphoproteomics workflows involve the processing of a single biological sample or a mixture of several biological samples in an individual vial (or a well in a plate) and the sequencing of them in a single LC-MS/MS run. The high sample throughput, robustness and ease-of-handling enable measurements of many samples, such as time-series measurements, making this approach increasingly popular. Additionally, single-run workflows have increased sensitivity over complex workflows because peptides are not divided over multiple fractions. However, the limited phosphoproteome depth and the presence of many phosphosites with missing quantification values across samples processed through single-run workflows compromise sample comparison. A recent study described an EasyPhos workflow for high-sample throughput without compromising the depth of the phosphoproteome coverage [8]. This workflow removed the early steps of the protocol involved in total peptide desalting before enrichment for phosphorylated peptides—a crucial element of its strategy. Recently, several additional improvements were made, including the elimination of a protein precipitation step, thus enabling samples to be processed in a single tube [9].

To study the impact of the sample preparation methods on phospho-signaling, we compared phosphoproteomes generated by two established workflows: fractionation-based [5,10,11,12] versus single-run [8,9,13,14,15] workflows. To this end, we exposed mouse embryonic stem cells (mESCs) to different doses of ionizing radiation (IR) and used either a complex workflow involving SCX-chromatography-based phosphoproteomics (SCXPhos) or the modified single-run high-throughput EasyPhos phosphoproteomics workflow (HighPhos). IR-induced DNA damage triggers the activation of an extensive network of cellular responses termed the DNA damage response (DDR) that involves the initial recognition of DNA damage by sensor proteins followed by the initiation of a vast network of signaling cascades mediated by an array of protein PTMs. While several PTMs are involved in the DDR, protein phosphorylation signaling plays a pivotal role in the DDR activation as it can rapidly control the function of proteins by altering their stability, activity, subcellular localization and protein–protein or protein–DNA/RNA interactions. Protein kinases and phosphatases (de)phosphorylate numerous substrates, often in a cascading manner, as several kinases/phosphatases themselves are substrates of other kinases/phosphatases. Three phosphoinositide 3-kinase (PI3K)-related kinases (PIKKs)—ATM, ATR and DNA-PKcs—play a pivotal role in the DDR by guiding the activation of several cellular and molecular responses depending on the type and the extent of DNA damage. Datasets from both sample preparation methods achieved an approximately equal coverage of around 20,000 phosphosites each. Comparison of the respective phosphoproteomes revealed method-specific quantification of most IR-responsive phosphosites. We discuss the potential origins of such method-specific identification of phosphosites and how to decrease biases in phosphoproteomics. Additionally, we briefly address method-specific biology in the context of the cellular response to IR. Finally, we describe the impact of the employed phosphoproteome methods on uncovering a broader spectrum of phosphorylation signaling biology after ionizing radiation.

## 2. Materials and Methods

### 2.1. Cell Culture, Protein-Labeling, and Cell Treatment

J1, B4418 and E14.IB10 mouse embryonic stem cells (mESC) (Cat# ATCC^®^ SCRC-1010™; LGC Standards GmbH, Wesel, Germany) were cultured in 10 cm dishes that were pre-coated with 0.1% (*w*/*v*) gelatin. Cells were grown, maintained and SILAC-labeled as previously described [12]. DNA damage was inflicted by exposing cells to 0.1Gy of X-rays (dose-rate 0.1 Gy/min) for low dose (LD; 2–4 DSBs per cell) and to 1 Gy (dose-rate 1 Gy/min) for high dose (HD; 20–40 DSBs per cell) (Thompson and Limoli, 2000) using the YXlon X-ray generator (YXlon International GmbH, Hamburg, Germany 200 KV). Following SILAC labeling, cells were mock-treated or exposed to LD or HD, and cell lysates were prepared at 0.5 h and 4 h after irradiation.

### 2.2. Strong Cation Exchange Chromatography-Based Phosphoproteomics (SCXPhos) Sample Preparation and MS Measurements

For SCXPhos, protein sample collection, phosphopeptide enrichment and clean-up were performed as follows. Briefly, after treatment and incubation, cells were lysed on dishes with urea lysis buffer (8 M urea, 50 mM Tris pH 8.1, 75 mM NaCl, 1 mM MgCl_2_, 10 mM sodium pyrophosphate, 1 mM sodium fluoride, 1 mM β-glycerophosphate disodium salt pentahydrate, 500 U benzonase, 100 µL each of phosphatase inhibitor cocktails 2 and 3 and 1 tablet cOmplete^TM^ mini protease inhibitor cocktail (Cat# 11836170001, Roche, Amsterdam, The Netherlands)). Samples were centrifuged (13,000 rpm for 15 min) to pellet the cell debris and protein concentrations were determined (Qubit Invitrogen). In total, 5 mg of protein per triple-SILAC-sample was taken, multiplexed and processed as follows. Samples were first reduced (2.5 mM dithiothreitol (DTT) for 25 min at 60 °C), alkylated (7 mM iodoacetamide for 15 min at room temperature (RT) in dark), quenched (2.5 mM DTT for 15 min at RT) and diluted 8-fold with 25 mM Tris (pH 8.1) −1 mM CaCl_2_. Proteins were then digested with Trypsin/Lys-C mix (Cat# V5072, Promega, Leiden, The Netherlands) at a 1:100 final ratio per enzyme for 18 h at 37 °C, and the reaction was stopped by adding trifluoroacetic acid (TFA; 0.4% final concentration). Subsequently, samples were centrifuged (3200 rpm for 5 min) and the supernatants containing tryptic peptides were lyophilized. Peptides were desalted (0.1% acetic acid) on C18 cartridges (Sep-Pak Vac 1 mL, Waters) and then eluted (0.1% acetic acid (ACA) and 30% acetonitrile (ACN)), lyophilized and fractionated. The fractionation was performed on a 9.4 by 200 mm 5 µm particle polysulfoethyl A strong cation exchange (SCX) column (PolyLC) at 1 mL/min using a 70 min gradient from 0 to 75 mM KCl and for 38 min with 350 mM KCl in 5 mM KH_2_PO_4_ (pH 2.65) and 30% acetonitrile. For each experiment, 18 fractions were collected, lyophilized and dissolved in buffer A (300 mg/mL lactic acid, 80% ACN and 0.1% TFA). Phosphopeptide enrichment was performed by loading the samples onto Titanium dioxide (TiO_2_) columns (TopTip 1–100 µL; Cat# TT1TIO96, Glygen Corporation, Columbia, MD, USA) prewashed with elution buffer (15 mM NH_4_OH, pH 10.5), equilibration buffer (0.1% TFA) and buffer A. The columns were then washed with buffer A, followed by buffer B (80% ACN (*v*/*v*), 0.1% TFA (*v*/*v*)) and phosphopeptides were eluted from the columns with elution buffer and collected in Eppendorf tubes with equal volumes of 2% TFA. Phosphopeptides were desalted (0.1% TFA) using Stage Tip C18 columns (Proxeon, Odense, Denmark) (pre-equilibrated with methanol, buffer B and 0.1% TFA), eluted (buffer B), lyophilized and stored at −80 °C until MS analysis.

### 2.3. High-Performance Liquid Chromatography (HPLC) and MS Measurements of Fractionated Samples

Phosphopeptides (5 µL/10 µL) were loaded onto a 15 cm column packed in-house with 1.9 µm C18 ReproSil particles (Cat# r119.aq, Dr. Maisch GmbH, Ammerbuch, Germany). An Easy-nLC 1000 system (Cat# LC140, Thermo Fisher Scientific, Odense, Denmark) coupled to the MS (Q Exactive, Thermo Fisher Scientific, Bremen, Germany) was used to separate the (phospho)peptides with a binary buffer system of buffer A (0.1% formic acid) and buffer B (60% acetonitrile, 0.1% formic acid) at a flow rate of 300 nl/min. Phosphopeptides were introduced into the column with buffer A and eluted with 5 to 25% of buffer B, resulting in a 2 h gradient.

We used a data-dependent Top10 MS acquisition method in which one full scan (400–2000 m/z, MS resolution 70,000 at 200 m/z) at a target of 3 × 10^6^ ions was performed, followed by 10 data-dependent MS/MS scans with higher-energy collisional dissociation (HCD) with the following parameters: a target of 1 × 10^5^ ions, maximum ion fill time 80 ms (phosphopeptides), isolation window 2.2 m/z, normalized collision energy (NCE) of 25% and underfill ratio of 1% at MS/MS resolution of 17,500 at 200 m/z. Dynamic exclusion of 60 s was enabled.

### 2.4. High Throughput Phosphoproteomics (HighPhos) Sample Preparation

Samples were prepared as described previously [8] with modifications as follows. After the treatment and incubation of cells, the medium was decanted, and the dishes were kept on ice. Cells were washed once with 10 mL ice-cold DPBS, collected by scraping and lysed in 1 mL HighPhos lysis buffer (6 M GdmCl (Guanidine hydrochloride) (Cat# G3272, Sigma, Amsterdam, The Netherlands), 100 mM Tris pH 8.5, 10 mM TCEP (Tris(2-carboxyethyl) phosphine) (Cat# C4706, Sigma, Amsterdam, The Netherlands), 40 mM CAA (2-Chloroacetamide) (Cat# 22790, Sigma, Amsterdam, The Netherlands) and 2 μL benzonase (250 U/μL) per 10 mL of lysis buffer (Cat# 71205-3, Novagen, VWR, Amsterdam, The Netherlands)). Lysates were snap-frozen in liquid nitrogen and stored at −80 °C until use. For phosphoproteomics, lysates were thawed and incubated on a roller bench at room temperature for 30 min. Protein concentration was measured in triplicate using a Pierce™ BCA Protein Assay Kit (Cat# 23225, Thermo Scientific, VWR, Amsterdam, The Netherlands). Per SILAC condition, 500µg of proteins were multiplexed in a 50 mL tube, heated to 95 °C for 5 min and cooled on ice for 15 min. They were then sonicated in a Bioruptor (Diagenode, Seraing, Belgium) at 4 °C for 10 min (sonication cycle: 30 s ON and 30 s OFF) and heated to 95 °C for 5 min and cooled on ice for 10 min. Lysates were then centrifuged at 13,000 rpm for 30 min at 4 °C; any residual cell pellets found were taken along with the supernatant and each tube was made up to 4 mL total volume with SuperQ. In total, 4x volumes of −20 °C acetone (Cat# 650501, Sigma, Amsterdam, The Netherlands) was added to the lysates, and proteins were precipitated overnight at −20 °C. Protein precipitates were collected by centrifugation at 10,000 rpm for 20 min at 4 °C and the pellets were washed twice with 2 mL of 80% acetone. Washed pellets were air-dried overnight. Protein pellets were dissolved in 500 μL of TFE digestion buffer (10% trifluoroethanol (TFE) (Cat# 05841-50 mL, Sigma, Amsterdam, The Netherlands) and 100 mM ammonium bicarbonate (ABC) (Cat# 09830, Sigma, Amsterdam, The Netherlands) in SuperQ) and transferred to a 96-well 2 mL deep well plate (DWP) (Cat# 0030 504.305, Eppendorf, VWR, Amsterdam, The Netherlands). In total, 1 μL of 1% ProteaseMAX detergent (Cat# V2071, Promega, Leiden, The Netherlands) in 50 mM ABC was added to each well of the DWP. Trypsin/Lys-C Mix (Cat# V5072, Promega, Leiden, The Netherlands) was added at a 1:25 ratio (1:50 end enzyme-to-protein ratio) to each well, sealed tight with a silicone mat (Cat# 951030147, Eppendorf, VWR, Amsterdam, The Netherlands), and the digestion was performed in a ThermoMixer C (Cat# 5382000015, Eppendorf, VWR, Amsterdam, The Netherlands) at 2000 rpm for 18 h at 37 °C.

Phosphopeptide enrichment was carried out as previously described [8] with minor modifications. Briefly, protein digestion was stopped by adding 150 μL of 3.2 M KCl, 55 μL of 150 mM KH_2_PO_4_, 800 μL of ACN and 95 μL of TFA to each sample, mixed at 2000 rpm, centrifuged to clear debris and the supernatants transferred to a new DWP. A total of 100 µL of TiO2 (Cat# 5010-21315, GL Sciences, Eindhoven, The Netherlands) bead suspension (10:1 beads/protein ratio) in loading buffer (80% ACN, 6% TFA) was added per well, incubated at 40 °C for 5 min at 2000 rpm. Beads containing phosphopeptides were centrifuged at 2000 rpm for 1 min and the supernatant was discarded. Beads were first transferred to a clean DWP by resuspending them in 2 × 500 μL of wash buffer (60% ACN, 1% TFA) and were then washed five times with 1 mL of wash buffer. Next, the elution of phosphopeptides was performed by transfer of the beads (suspended in 100 μL of transfer buffer (80% ACN, 0.5% acetic acid)) onto the top of a C8 StageTip, and centrifugation was performed until no liquid remained. Subsequently, 30 µL of elution buffer (40% ACN, 15% NH_4_OH (25%, HPLC grade)) was added and was followed by centrifugation to collect the samples in PCR plates (Cat# AB-1300, Thermo Scientific, VWR, Amsterdam, The Netherlands) containing 15 µL of 10% TFA. Phosphopeptides were then desalted using SDB-RPS StageTips (3× layers; (Cat# 66886-U, Sigma, Amsterdam, The Netherlands)) pre-equilibrated with 0.2% TFA, washed two times with 0.2% TFA and eluted with 60 µL of SDB-RPS elution buffer (80% ACN, 5% NH_4_OH (25% HPLC grade)) into a clean auto-sampler plate. Phosphopeptides were then lyophilized to complete dryness and kept frozen at −80 °C until MS measurement.

### 2.5. HighPhos HPLC and MS Measurements

Before MS measurement, phosphopeptides were resuspended in 0.1% formic acid and were subsequently loaded (9 μL/10 µL) onto a 50 cm column with a 75 µm inner diameter, self-packed with 1.9 µm C18 ReproSil particles (Cat# r119.aq, Dr. Maisch GmbH, Ammerbuch, Germany) maintained at 50 °C. A nano-liquid chromatography (Easy-LC 1200 system; Cat# LC140, Thermo Fisher Scientific, Odense, Denmark) coupled to a mass spectrometer (Q Exactive HF; Thermo Fisher Scientific, Bremen, Germany) was used to separate the phosphopeptides with a binary buffer system of 0.1% formic acid (Buffer A) and 80% ACN (Buffer B) at a flow rate of 250 nl/min. Phosphopeptides were eluted with a 4.5 h gradient consisting of 25% buffer B for 220 min, 60% buffer B for 35 min and 5% buffer B for 10 min. Instruments and systems were obtained from Thermo Fisher Scientific unless stated otherwise.

We performed a data-dependent Top10 MS acquisition method using one full scan (300–1750 m/z, MS resolution 120,000) and set a target of 3 × 10^6^ ions. Next, the ions were fragmented with higher-energy collisional dissociation (HCD) and analyzed by data-dependent MS/MS scans with the following parameters: a target of 2 × 10^5^ ions, maximum ion fill time 108 ms, isolation window 1.3 m/z, normalized collision energy (NCE) of 28% at MS/MS resolution of 60,000. Dynamic exclusion of 20 s was enabled to prevent repeated sequencing of peptides.

### 2.6. MS Bioinformatics and Data Analysis

The raw MS data were processed using MaxQuant (v 1.5.7.4) [16] and default settings with the following modifications. SILAC triplet was activated (Light = default; Medium = Lys4; Arg8; Heavy = Lys8; Arg10). Acetylation (Protein N-term; +42.010565 Da), oxidation (M; +15.994915 Da) and phosphorylation (STY; +79.966330 Da) were selected as variable modifications, and carbamidomethylation (C; +57.021464 Da) was selected as a fixed modification. LysC/P and Trypsin/P were selected for enzymes. A minimum peptide length of 6 amino acids was set, and “cut peaks”, “advanced peak splitting” and “match between runs” (MBR) were enabled with the default values (alignment time window: 20 min and matching time window: 0.7 min). The “Filter labeled amino acids” parameter was disabled, while the “re-quantify”, “second peptides” and “advanced ratio estimation” parameters were enabled. A protein-, peptide- and site-level FDR of 0.01 was applied using a target-decoy approach with a reverse sequence database generated automatically by MaxQuant. Proteins, peptides and sites were identified by the inbuilt Andromeda search engine using the following four mouse FASTA databases: protein-coding transcript translation sequences (Gencode v12; August 2016), mitochondrial proteins (MitoCarta 2.0) [17], alternative proteins translated from alternative open reading frames (mouse AltORFs) [18] and Uniprot canonical isoform proteins (December 2016). Further downstream analyses were performed using Perseus (v1.5.5.3) [19], Microsoft Excel (2016), R (v4.1.0), R studio (v1.4.1103; ggplot2, ComplexHeatmap [20] and circlize packages), Python (v3.9.6; NumPy and Pandas packages) and GraphPad Prism (v7.02); protein annotations were extracted from Gene Ontology (GO) and kinase–substrate relations were based on the PhosphositePlus database (phosphosite.org, accessed on 15 April 2021).

For further data processing, normalized SILAC ratios from all samples were first log2 transformed after filtering for valid values, removing contaminants and reverse hits. We also removed H/M SILAC ratios from further data processing steps after reporting the total number of quantified phosphosites since, throughout our analysis presented here, we compare relative changes over untreated controls that were included in all experiments as Light SILAC labels. For phosphoproteome data, the site table was expanded to obtain singly, doubly and multiply phosphorylated sites.

### 2.7. Statistics

**Figure 1:**
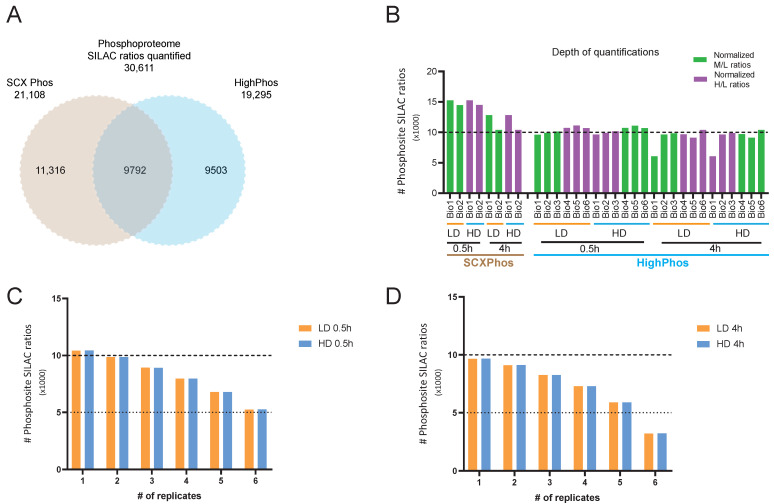

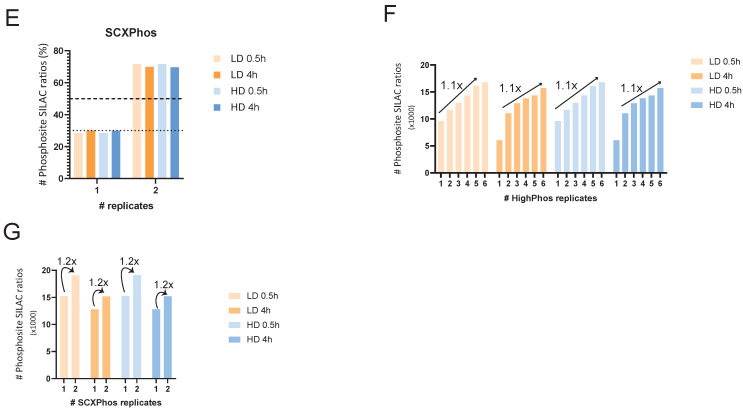
Comparative analysis of SCXPhos and HighPhos. (**A**) Venn diagram depicting the overlap of total phosphosite SILAC ratios (PSRs) quantified in SCXPhos (brown circle) and HighPhos (blue circle) datasets of J1 mESC. (**B**) Bar chart depicting total number of PSRs quantified in each phosphoproteome experiment. Normalized PSRs quantified as M/L and H/L ratios are indicated by green and purple bars. Bar chart of replicate-wise quantified PSRs in each HighPhos experiment after LD (orange bars) and HD (blue bars) at (**C**) 0.5 h and (**D**) 4 h after IR exposure. *Y*-axis represents number of PSRs quantified in one, two or all six replicates (*X*-axis). (**E**) Bar chart depicting the percentage of PSRs quantified in one or both replicates within SCXPhos experiments. Bar charts depicting progressively increasing number of PSRs quantified in (**F**) six replicates of HighPhos experiment and (**G**) two replicates of SCXPhos.

We designed SCXPhos and HighPhos experiments in such a way to achieve an equivalent depth of the total quantified phosphosites. We performed SCXPhos (18 fractions × 2 h gradient per fraction) in duplicate and HighPhos (single-run 4.5 h gradient per sample) in six replicates. For SCXPhos, we defined the dynamic phosphoproteome response by applying either a significance A test (Benjamini-Hochberg FDR < 0.05) to both biological replicates or passing a 1.5-fold change threshold filter in both biological replicates in any experimental condition. Since, on an average, 70% of the phosphosites in SCXPhos were quantified in both replicates (Figure 1G), we aimed to achieve a similar quantification overlap in the HighPhos experiment. We found that approximately 77% of the phosphosites in the HighPhos dataset were quantified in four out of six biological replicates (Figure 1E,F). Additionally, we aimed to identify the equivalent percentage of IR-responsive phosphosites from both experiments to enable direct comparison and found ANOVA statistical testing of the HighPhos dataset to be optimal to reach such a criterion. Hence, for HighPhos, significantly responding phosphosites were called by Analysis of Variance (ANOVA; FDR < 0.05) or phosphosites passing a 1.5-fold change threshold in 4/6 biological replicates.

### 2.8. Large-Scale In-House Generated Phosphoproteome Datasets

We generated phosphoproteomes of E14.IB10 mES cells after exposure to LD and HD at nine different time points (5 s, 30 s, 5 min, 30 min, 1 h, 2 h, 4 h, 8 h and 24 h) in six biological replicates representing 162 global phosphoproteomes. Next, we generated two DNA double-strand break (DSB) repair deficient cell lines by knocking out key proteins (LIG4 and KU80) in the non-homologous end joining (NHEJ) pathway by CRISPR-Cas9 gene editing. We then generated phosphoproteomes of these two mutant cell lines after exposure to LD and HD at six different time points (0.5 h, 1 h, 2 h, 4 h, 8 h and 24 h) in four biological replicates, each representing 144 global phosphoproteomes. Taken together, 306 global single-run IB10 phosphoproteomes were generated using the HighPhos method described above. For the SCXPhos datasets, we used B4418 cell lines to profile the phosphoproteome at 4 h after LD and HD IR exposures.

### 2.9. Cloud-Enabled High-Performance Computing (cHPC) and Bioinformatics

Cumulative phosphoproteome analysis (CPA) was performed in a cHPC cluster (SurfSara). Briefly, previously published datasets [8,14,15] and a large-scale in-house generated dataset (described above; Sampadi et al., manuscript in preparation) were downloaded onto the cloud server and searched using MaxQuant in a Windows 10 virtual machine with 64 cores and 150GB RAM. In total, 893 RAW files were used for the CPA analysis. Among these 893 RAW files, 437 files involving our in-house generated datasets were all triple SILAC-labeled, whereas the remainder (456) of the raw files obtained from published studies used for the comparison were label-free experiments. Over 570 of the 893 RAW files involved single-run analyses, whereas the remainder (323) represented fractions of SCXPhos samples. We performed the CPA analysis on two groups of raw files—570 single-runs (comprising 114 in-house SILAC and the remainder 456 published label-free) and 323 SCX fractions (all in-house SILAC) were grouped separately and given different unrelated fraction numbers, and hence no matching was performed between these two sets of samples. We measured most 323 SCXPhos RAW files using 2 h gradients with identical column dimensions and same buffers. All 570 single-run samples were measured over 4.5 h gradients using columns with similar dimensions and particles (75 μM inner diameter, packed in-house with 1.9 μM C18 ReproSil particles (Dr Maisch GmbH) and maintained at 50 °C using a column oven) and similar buffers. Matching between SILAC and label-free single-runs (exact same gradients, column and buffers) was performed with the assumption that SILAC inherently contain “label-free” measurements in the “Light” SILAC channel in all experiments, and hence, matching between these samples would be appropriate as the MBR algorithm relies also on exact masses apart from other criteria. However, the MBR step does not include an inherent FDR statistical control step and may result in the formation of some false positives.

Searches were performed by grouping SILAC samples and label-free samples as separate groups with either SILAC labels turned ON or OFF, respectively. For SILAC samples, SILAC triplet was activated (Light = default; Medium = Lys4; Arg8; Heavy = Lys8; Arg10). For all RAW files, acetylation (Protein N-term; +42.010565 Da), oxidation (M; +15.994915 Da) and phosphorylation (STY; +79.966330 Da) were selected as variable modifications, and Carbamidomethylation (C; +57.021464 Da) was selected as a fixed modification. LysC/P and Trypsin/P were selected for enzymes. A minimum peptide length of 6 amino acids was set; “cut peaks”, “advanced peak splitting” and “match between runs” (MBR) were enabled with the default values (alignment time window: 20 min and matching time window: 0.7 min). The “Filter labelled amino acids” parameter was disabled, while the “re-quantify”, “second peptides” and “advanced ratio estimation” parameters were enabled. A protein-, peptide- and site-level FDR of 0.01 was applied using a target-decoy approach with a reverse sequence database generated automatically by MaxQuant. Proteins, peptides and sites were identified by the inbuilt Andromeda search engine using the following four mouse FASTA databases: protein-coding transcript translation sequences (Gencode v12; August 2016)), mitochondrial proteins (MitoCarta 2.0) [17], alternative proteins translated from alternative open reading frames (mouse AltORFs) [18] and Uniprot canonical isoform proteins (December 2016).

### 2.10. Pathway Analysis

The identification of enriched pathways from responsive phosphosites was achieved by the Reactome pathways (*p*-value/FDR < 0.05; https://reactome.org/, accessed on 22 April 2021).

## 3. Results

### 3.1. Quantitative Phosphoproteomics

Phosphoproteomes of SILAC-labeled mouse embryonic stem cells (mESCs) were generated after exposure to ionizing radiation (Figure S1A). SILAC media containing isotopologues of light (Arg0 Lys0; L), medium (Arg6 Lys4; M) and heavy (Arg10 Lys8; H) amino acids were used to propagate mESCs over seven passages to achieve complete protein labeling. SILAC-labeled cells were mock-treated or exposed to 0.1 Gy (LD) and 1 Gy (HD) of X-rays, and cell lysates were prepared at 0.5 h and 4 h after irradiation (Figure S1B). L, M and H SILAC-labeled lysates per experiment were mixed in equal ratios based on protein amount and digested into peptides with Lys-C/Trypsin mix. We used two different experimental setups to identify IR-induced alterations in the phosphorylation status of proteins. In one set of experiments, we used a strong cation exchange (SCX) chromatography fractionation-based phosphoproteomics (SCXPhos) method, while in another set of experiments, we applied a single-run high-throughput EasyPhos phosphoproteomics (HighPhos) method. To prevent bias originating from the protein digestion step or enrichment material used, we applied identical conditions for both phosphoproteomics methods regarding the digestion of proteins with the Lys-c/Trypsin enzyme mix (except the trypsin–protein ratio) and enrichment for phosphopeptides using titanium dioxide (TiO2) beads. For SCXPhos, peptides were cleaned up using solid-phase extraction (SPE) using C18 cartridges and fractionated into 18 fractions using SCX chromatography; subsequently, we performed phosphopeptide enrichment in each fraction using TiO2 beads to obtain phosphopeptides. Each fraction containing phosphopeptides was then cleaned up using C18 Stage Tips and analyzed using a Q Exactive mass spectrometer (QE MS). For HighPhos, phosphopeptide enrichment using TiO_2_ beads was performed directly on digested peptides without prior fractionations in parallel for all samples. Each phosphopeptide sample was cleaned using SDB-RPS Stage Tips and analyzed using a Q Exactive HF mass spectrometer (QE-HF MS).

### 3.2. Improvements to Phosphoproteome Sample Preparation

The original EasyPhos method [8] used the powerful chaotropic agent guanidine hydrochloride (GdmCl) to solubilize proteins. We opted for the addition of benzonase to the GdmCl buffer to digest nucleic acids and hence to release chromatin-bound proteins, reduce the viscosity of lysates and prevent insoluble pellet formation during lysis. Indeed, we consistently found only tiny cell pellets after the centrifugation of lysates treated with benzonase. A previous study reported that in global MS-based proteomics experiments, the number of protein identifications could be significantly increased by omitting cell pellet removal during trypsin digestion or adding ProteaseMAX detergent [21]. We reasoned that improved protein identification will also be beneficial for phosphoproteomics experiments, as the initial cell lysate preparation and protein digestion steps are the same for both proteomics and phosphoproteomics experiments. Hence, we omitted the centrifugation step before protein digestion and added ProteaseMAX detergent enhancer to each sample to improve the efficiency of protein digestion. Protein digestion was performed by adding Lys-C/Trypsin mix in a 1:25 (enzyme:protein) ratio (the final ratio for each enzyme was 1:50) and incubating samples for 18 h at 37 °C. Next, phosphopeptide enrichment was performed, and samples were sequenced by QE-HF MS (Figure S1). We performed a label swap experiment with six biological replicates: (i) in one set comprising three biological replicates, L, M and H SILAC-labeled cells were mock-treated, LD- and HD-exposed, respectively, (ii) in the other set comprising three different biological replicates, we kept L SILAC-labeled cells as mock-treated; however, we exposed M and H SILAC-labeled cells to HD and LD, respectively (Figure S1B). For SCXPhos, we used Lys-c/Trypsin mix at a 1:100 ratio and performed the experiments in two biological replicates, in each of which L, M and H SILAC-labeled cells were mock-treated, LD-exposed and HD-exposed, respectively (Figure S1), and samples were sequenced using QE-MS. SILAC was used for protein labeling as it is one of the most accurate quantification techniques for phosphoproteomics [22]. SILAC also enables small-scale multiplexing and thus a robust ratio estimation across many measurements over unirradiated controls (unirradiated controls are included in the light SILAC channel of all experiments). However, SILAC labeling results in repeated sequencing of phosphopeptide isotopologue variants in MS, leading to a decreased total number of phosphosite SILAC ratio (PSR) quantifications, and contributes to a relatively large number of missing quantification values in phosphoproteome datasets [22]. Despite such limitations, this strategy of HighPhos workflow enabled a sufficient coverage of the phosphoproteomes quantified, reaching around 10,000 PSRs per sample (see below), whereas for phosphoproteomics experiments, label-free experiments have an apparent lower precision; TMT labeling resulted in lower accuracy [22] despite achieving an apparent increase in precision and thus identifying more responsive phosphosites than SILAC. However, we chose to use SILAC as the best compromise when comparing the following aspects: quantification accuracy, precision, coverage and moderate throughput.

### 3.3. Comparative Analysis of SCXPhos and HighPhos Phosphoproteomes

**Figure 2:**
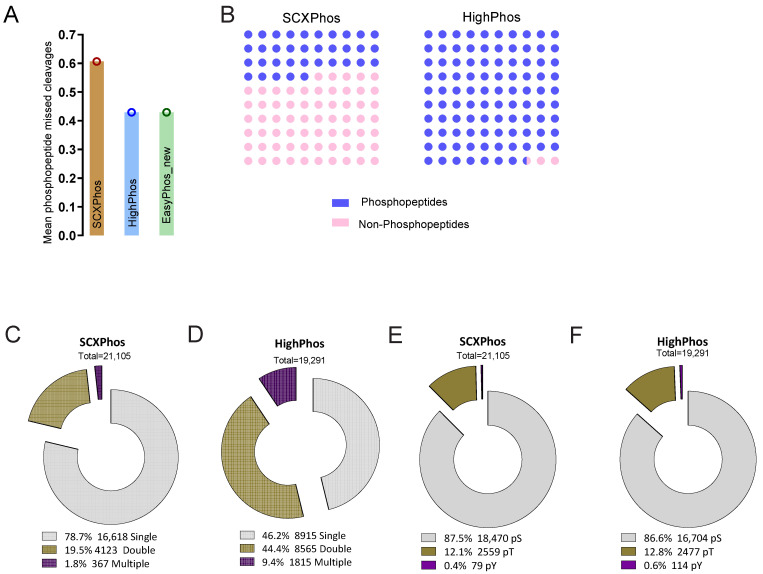
Comparison of SCXPhos and HighPhos datasets. (**A**) Bar charts depicting the phosphopeptide digestion efficiency of SCXPhos (brown bar), HighPhos (blue bar) and EasyPhos_new (green bar). The data for EasyPhos_new (green bar) represent “match all” data from Figure S2A of Humphrey et al., 2018. (**B**) 10 × 10 dot plot depicting the percentage of quantified phosphopeptides (blue) and non-phosphopeptides (light pink) for SCXPhos (left panel) and HighPhos (right panel). (**C**,**D**) Exploded donut plots of phosphorylation multiplicity within SCXPhos and HighPhos datasets. Total numbers and percentages of PSRs containing single, double, and multiple phosphorylation events are indicated per dataset. (**E**,**F**) Exploded donut plots of amino acid distributions within SCXPhos and HighPhos datasets. Total numbers and percentages of PSRs containing phosphorylated serine (pS), threonine (pT) or tyrosine (pY) residues are indicated per dataset.

To directly compare the phosphoproteomes quantified by the two methods—SCXPhos and HighPhos—we performed a MaxQuant search with all raw MS files in a single search. This search resulted in the quantification of 30,611 PSRs in total (Supplementary Table S1). Out of these, 21,108 and 19,295 PSRs were quantified by SCXPhos and HighPhos, respectively. Over 9792 PSRs (32%) were quantified by both methods, while 11,316 (37%) and 9503 (31%) PSRs were exclusively quantified by SCXPhos and HighPhos, respectively (Figure 1A). PSR quantification depth comparison revealed that 10,000–15,000 PSRs were quantified for each biological replicate by SCXPhos and 9000–11,000 by HighPhos, except for one sample (4 h Bio1) reaching 6000 (Figure 1B). More than 70% of the PSRs were quantified (Figure 1C,D) in at least four out of six biological replicates in the HighPhos experiment. Similarly, around 70% of the PSRs were quantified in both biological replicates of the SCXPhos experiments (Figure 1E). With increasing numbers of biological replicate measurements, the total number of quantified PSRs increased steadily for both HighPhos and SCXPhos (Figure 1F,G). Next, we compared the two procedures on the effectiveness of phosphopeptide digestion, enrichment and stoichiometry. We observed an improved phosphopeptide digestion efficiency with HighPhos compared to SCXPhos, with fewer missed cleavages. These findings are on par with a recent update to the EasyPhos protocol [9] (Figure 2A). While HighPhos enriched phosphopeptides with very high selectivity (on average > 97% across all samples), SCXPhos achieved on average of only about 35% enrichment (Figure 2B). Overall, despite using 10 times less protein input and 3 times less MS time, HighPhos was able to achieve a similar phosphoproteome coverage to SCXPhos and allowed quantification in six biological replicates, increasing the statistical power of the dataset. While the PSRs from the SCXPhos method contained a large proportion (around 78%) of single phosphorylation events (Figure 2C), the PSRs from the HighPhos method contained almost equal proportions of single (around 46%) and double (around 44%) phosphorylation events (Figure 2D). HighPhos also profiled a larger number of multi-site phosphorylation events (around 9%) compared to SCXPhos (around 1%). However, the stoichiometry of the serine, threonine and tyrosine phosphorylation events was highly similar for both methods (Figure 2E,F). Notably, we achieved a deeper coverage of double and multi-site phosphorylation events from HighPhos experiments, as evident from the numbers of phosphosites quantified being equivalent to those studies that profile very deep phosphoproteomes (>40,000 sites) employing complex fractionation and enrichment strategies [23].

### 3.4. Cumulative Phosphoproteome Analysis

Subsequently, we employed an exploratory analysis called cumulative phosphoproteome analysis (CPA) with the following goals in mind:

(1). To explore the total number of phosphosites that can be identified across samples; (2). To determine whether MBR across several RAW files dramatically increase the PSR quantifications; (3). To compare the overlap of PSRs between SCXPhos and HighPhos using different cell lines. (4). To determine the influence of a very large-scale dataset on the method-specific sites identified in the study.

In this CPA strategy, we analyzed the publicly available mouse EasyPhos datasets [8,14,15] together with a large-scale in-house raw dataset (see methods). We analyzed 893 raw MS files representing >800 biologically distinct phosphoproteomes together in a single MaxQuant search and utilized the match between runs (MBR) feature to transfer identifications between phosphoproteomes (Figure S2A). We performed a single MaxQuant run with all raw files to enable a direct comparison between various datasets. As this task was computationally intensive, we made use of the SurfSara High-Performance Computing Cluster (Dutch public service). We set up a Windows 10 virtual machine (VM) with 64 cores and 150GB RAM for this analysis. The search spanned over 35 days with an overall computational time of around 54 × 10^3^ CPU h. This search resulted in the quantification of >82,000 phosphosites. The results revealed that the number of PSRs quantified in SCXPhos experiments increased by 200, whereas those quantified in HighPhos experiments increased by over 2000 (Figure S2B; Supplementary Table S2), suggesting a beneficial use of CPA in HighPhos experiments to identify additional PSRs. The moderate increase of 2000 PSRs is comparable to published studies that do not use a large number of files for MBR [9] and hence does not appear to inflate false positive rates (Figure S2C). However, one should keep in mind that the MBR step in MaxQuant v1.5.7.4 used in this study inherently does not include an FDR statistical control step and should be cautious in interpreting the CPA results.

The correlation of four discrete time-point samples measured using Q Exactive (QE) with that of four different time-points measured using Q Exactive HF (QE-HF) orbitrap mass spectrometers unveiled a large overlap in PSR quantifications between these two instruments. Of the total 27,016 PSRs quantified by both instruments, 57% (15,392/27,016 PSRs) were quantified by both machines, whereas 2538 (9%) and 9086 (34%) PSRs were quantified uniquely in samples analyzed by QE and QE-HF, respectively (Figure S2D). In other words, around 86% (15,392/17,930) of PSRs quantified by QE measurements were also quantified by QE-HF measurements, while QE-HF measurements quantified an additional 35% (9086/27,016) PSRs. However, the distribution of single/double/multiple sites are equivalent in QE-specific, QE-HF-specific and shared sites (Figure S2E), suggesting that the contribution of different mass spectrometers in the biological differences are minimal.

The comparison of SCXPhos and HighPhos datasets of different mESC lines (B4118 and J1) generated in-house (see methods) revealed an overlap of 33% (9204/27,965 PSR) (Figure S2F) which is equivalent to the levels obtained when comparing datasets that are obtained from the same cell line (Figure 1A and Figure S2B). Conversely, the comparison on phosphoproteomes from two different mESC lines (J1 and IB10) profiled using the same (HighPhos) method revealed a much larger overlap. Over 14,794 PSRs (52%) were quantified in both cell lines, while 7103 and 6813 PSRs were quantified exclusively in J1 and IB10 cell lines using the same HighPhos method (Figure S2G) (left panel). Similarly, in SCXPhos experiments, an overlap of 53% (13,177 PSRs) was obtained between the phosphoproteomes of two different cell lines (J1 and B4118) (Figure S2G) (right panel). These results contrast with the observed around 33% overlap between the phosphoproteomes of the same cell line that were obtained from two different methods (Figure 1A and Figure S2B). These findings suggest that the impact of the methodology on the uncovered phosphoproteome is greater than that originating from the use of different cell lines. Consistently, only a discrete overlap of 11,079 phosphosites (23%) (Figure S2H) was observed when comparing previously published SCXPhos and single-run phosphoproteome datasets of EGF-stimulated cells. The comparison of single-run phosphoproteomes of multiple mouse tissues uncovered that the fraction of tissue-specific phosphosites is strikingly small (Figure S2I). We found 0.2–9% of the phosphosites to be exclusively quantified in one of the compared tissues, with liver tissue showing the highest uniquely quantified phosphosites (8105; 9%). However, over 12,133 PSRs (15%) were exclusively quantified in mESCs despite a relatively lower coverage (48,102 sites) than the liver phosphoproteome (59,505). Over 55% (6605 PSRs) of these 12,133 PSRs were exclusively derived from the SCXPhos datasets (Figure S2I), providing direct evidence for the method-specific impact on phosphoproteome quantification. These results suggest that the application of different sample preparation methods may lead to the identification of more phosphosites than repeated sequencing using a single sample preparation method.

### 3.5. Identification of Method-Specific Signaling Biology

**Figure 3:**
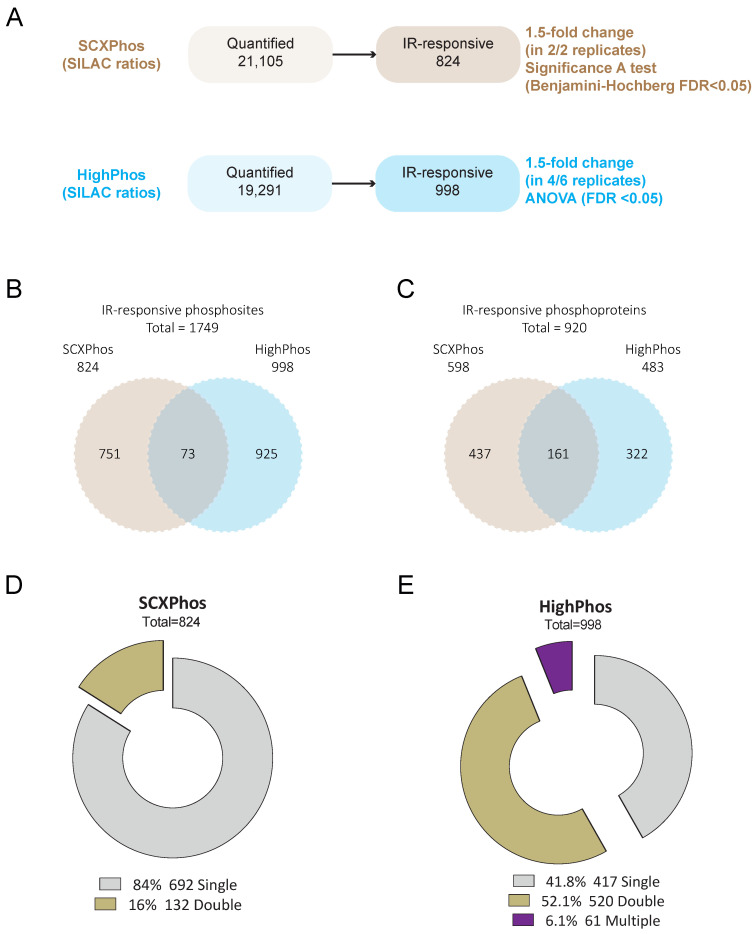
Overview of PSR statistics from SCXPhos and HighPhos. (**A**) Identification of IR-responsive PSRs from SCXPhos (top panel) and HighPhos (bottom panel). (**B**) Venn diagram depicting the overlap of IR-responsive PSRs and (**C**) phosphoproteins from SCXPhos (brown circle) and HighPhos (blue circle). Total number of IR-responsive PSRs/phosphoproteins within each experiment is indicated. (**D**,**E**) Exploded donut plots of phosphorylation multiplicity within SCXPhos and HighPhos datasets. Total number and percentage of PSRs containing single, double, and multiple phosphorylation events are indicated per dataset. Total number of IR-responsive PSRs within each experiment is indicated.

Phosphosites/phosphoproteins: SCXPhos uncovered 824 PSRs on 598 proteins to be responsive upon IR exposure, whereas HighPhos uncovered over 998 IR-responsive PSRs on 483 proteins (Figure 3A). Taken together, we identified over 1749 PSRs on 920 proteins that were IR-responsive, of which over 96% were method specific (Figure 3B; Supplementary Table S3). Over around 58% (581/998) of the IR-responsive PSRs from HighPhos concerned double- (520 sites) and multi-site (61 sites) phosphorylation events (Figure 3C). In contrast, only around 16% (132/824) of the IR-responsive PSRs from SCXPhos contained double-site and none contained multi-site phosphorylation events (Figure 3D). Out of the 4% (73) IR-responsive PSRs that were commonly identified by both methods, 49 and 24 PSRs were singly and doubly phosphorylated, respectively. Similar trends were observed for the proteins to which these phosphosites belong to (phosphoproteins), with both methods identifying commonly responding phosphosites only on 161 proteins and the remainder being method specific (Figure 3C). Even among those 161 common phosphoproteins, only 52 contained shared identified IR-modulated phosphosites (73 PSRs) (Figure 3B). The remainder (109/161 phosphoproteins) contained different phosphosites for each method. Despite such a minimal overlap in PSRs and phosphoproteins, both methods enriched several well-known IR-induced pathways such as ATM, ATR, p53, DDR, cell cycle, apoptotic and pluripotency pathways (Figures S3 and S4).

**Figure 4:**
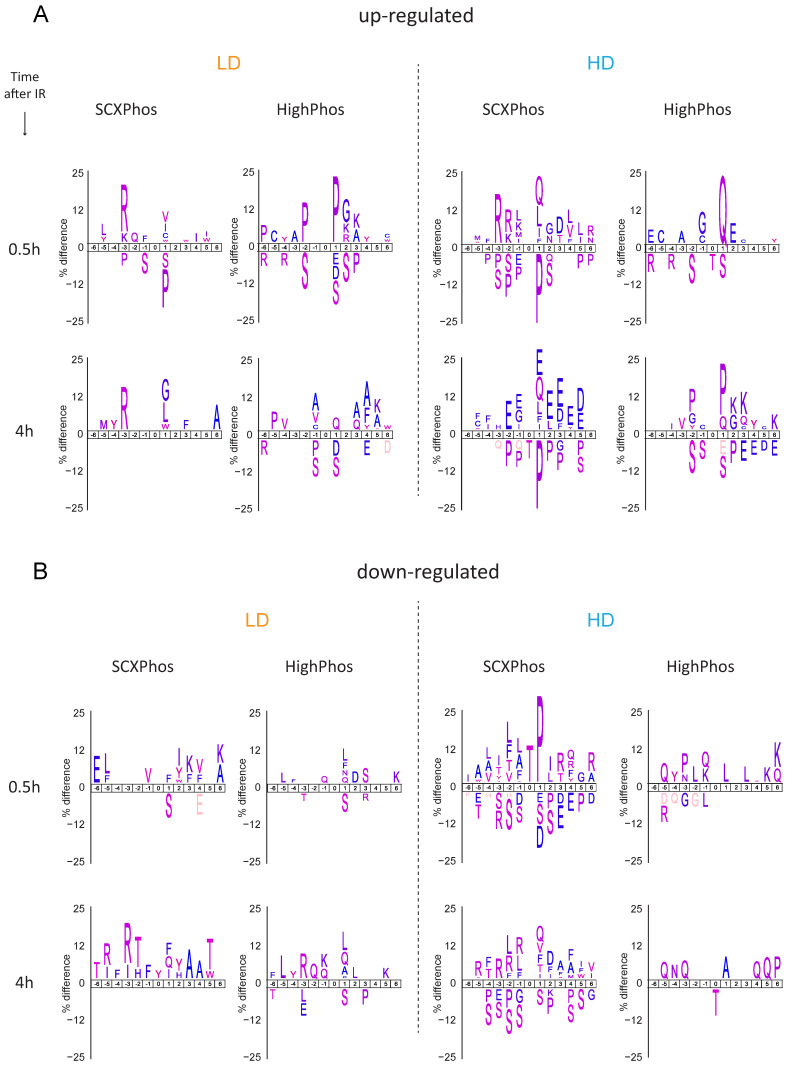
Motif analyses of differentially regulated PSRs. Sequence motifs were obtained from the IceLogo tool. For each condition, a statistical background of the sequences of all quantified phosphosites was used with a 0.05 *p*-value cut-off. Position 0 represents a phosphorylated amino acid residue. LD and HD refer to data obtained after exposure to low (LD) and high (HD) doses of ionizing radiation. Motifs enriched for each condition among the (**top panel**) up-regulated phosphosites and (**bottom panel**) down-regulated phosphosites.

**Figure 5:**
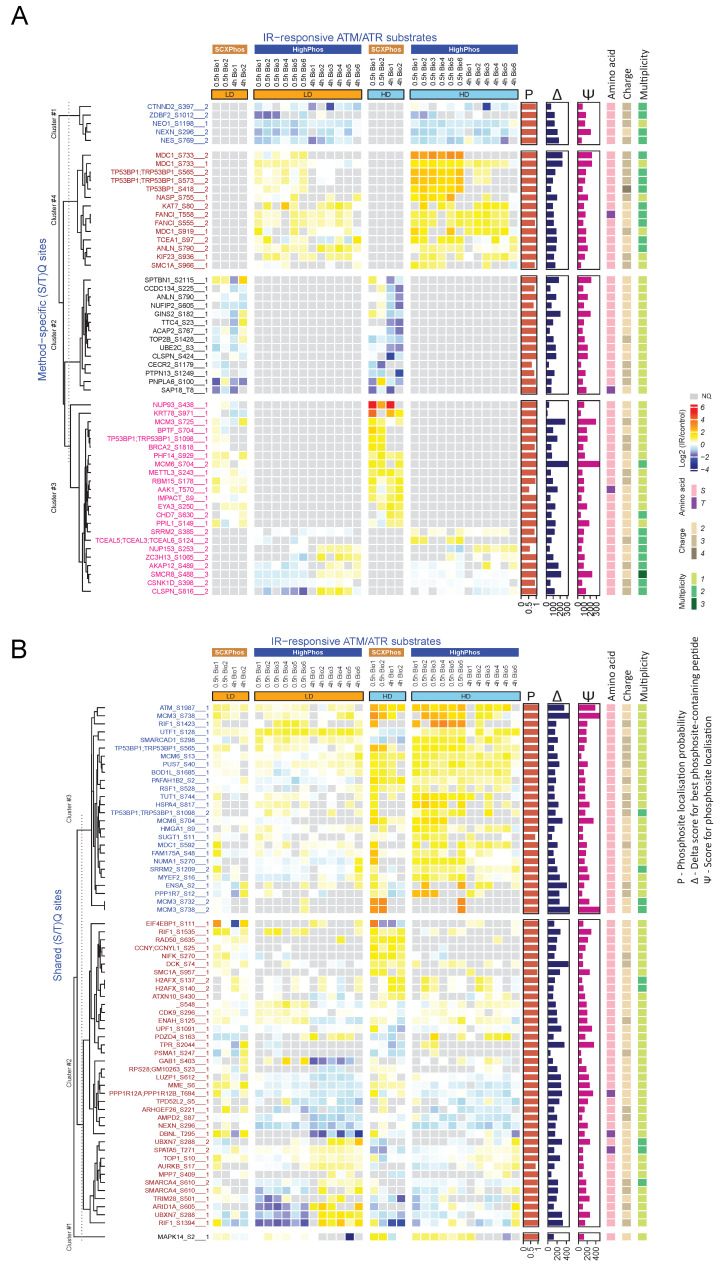
Heatmap of (S/T)Q motif-containing PSRs. Heatmap of the IR-responsive (S/T)Q motif containing PSRs: (**A**) Method-specific and (**B**) shared (S/T)Q phosphosites. Grey blocks indicate NQ (no quantifications). Hierarchical clustering was performed to order phosphosites. Phosphosites labels should be read as follows: Gene_phosphorylated amino acid phosphorylated residue_multiplicity. Multiplicity represents the number of phosphorylation events observed. For each phosphosite, the phosphorylated amino acid, and the number of phosphorylation events observed are annotated on the right side of the heatmap.

Kinase substrate motifs: The ATM/ATR kinase motif ((S/T)Q) was enriched among the up-regulated PSRs from both methods after HD exposure at both time points; however, the enrichment was most pronounced in the HighPhos experiment at 0.5 h (Figure 4). ATM/ATR substrate analyses showed most uncovered (S/T)Q sites to be method specific, with only a few substrates including ATM (S1987; autophosphorylation site) and 53BP1 being identified by both methods (Figure 5). Quantification plots of shared substrates, such as ATM (pS1987), 53BP1 (pS565), MCM3 (pS738), MCM6 (pS13), PUS7 (pS40) and TUT1 (pS744), revealed highly reproducible quantifications between both methods (Figure S5).

SCXPhos uniquely overrepresented the AKT (R-x-x-(S/T)), PKA ((R-R-x(S/T)) and PLK ((S/T)-(L/I/F)) kinase motifs, while HighPhos uniquely enriched the proline-directed kinase motif (P at +1 position) among the up-regulated PSRs after both LD and HD exposures (Figure 4). Whereas SCXPhos enriched acidic residues (D/E) at + 3 position, a motif for the CK2 kinase—a regulator of DNA repair—at 4 h after HD exposure, HighPhos showed a depletion for this motif at this time point (Figure 4). Additionally, SCXPhos enriched the PIM1 kinase ((S/T)G) motif among the up-regulated PSRs after LD exposure. Down-regulated PSRs from SCXPhos, but not from HighPhos experiments, showed a clear enrichment for both phosphorylated threonine residue and proline-directed motif at 0.5 h after HD exposure. At 4 h after both LD and HD exposure, responsive sites from both SCXPhos and HighPhos enriched the AMT/ATR and PLK kinase motifs, apart from several other unknown motifs. Meanwhile, the AKT kinase motif was overrepresented at 0.5 h after LD exposure in both methods.

Kinome/phosphatome: Congruently, kinome/phosphatome (K/P) analyses uncovered that both methods identified a unique set of IR-responsive K/P phosphosites with minimal overlap. Several K/Ps that respond to the production of reactive oxygen species (ROS) were enriched among the LD-exposed cells by both methods, identifying a complementary set of K/Ps with minimal overlap (Figure S6). While both methods commonly identified ROS-sensitive K/Ps after LD exposure, the identities of the K/Ps and their phosphorylation sites were dramatically different between the methods. In contrast, both methods identified a subset of shared DDR-related K/Ps, such as ATM pS1987, CTDSPL2 pS134 and MAPK14 pS2 after HD exposure, all displaying a similar trend in alterations. Consistently, ATM, p53 and DDR pathways were enriched among the up-regulated PSRs after HD exposure from both methods (Figures S7 and S8). Despite such dramatic differences in identified K/Ps, pathway analyses of K/Ps uncovered that the enriched biological pathways largely overlapped between these two methods.

## 4. Discussion

In this study, we performed a comparative analysis of phosphoproteomes from mESCs exposed to IR that were profiled using SCXPhos and HighPhos procedures. This analysis revealed the impact of applied phosphoproteomics methods on the quantified and IR-responsive phosphoproteomes generated. Although the collected datasets contained equivalent coverage of PSRs quantified (around 20,000 PSRs), the overlap between the methods was lower (9792 PSRs, 32%) (Figure 1A) and significantly smaller than the overlap (around 52%) (Figure S2G) when profiling two different cell lines using the same method at equal coverage. Combining the datasets dramatically increased the coverage by 50% to >30,000 total quantified PSRs.

Within both methods, the coverage of the phosphoproteome increased when more biological replicate measurements were included (Figure 1F,G), indicating that each measurement profiles a distinct subset of the phosphoproteome. The semi-stochastic nature of ion sampling in the MS contributes to this variability in phosphosites profiled and is presumably one of its primary sources [22]. Biological variations in signaling events might be another source for such differences but is likely to be a minor contributor, as becomes evident from the highly reproducible quantification of some key phosphosites in the DDR by both methods (Figure 5). The dramatic increase in the number of PSRs quantified when biological replicates are added from a different phosphoproteomics method (Figure 1A) when compared to replicate measurements within each method (Figure 1F,G) suggests that most differences in PSRs uncovered originate from sample preparation conditions rather than from biological variations or semi-stochastic ion sampling in MS. A typical source for such variation arising from MS is the sequencing speed of the MS instrument used—as we used QE-HF for HighPhos, which has increased ion isolation performance and faster ion scanning speed when compared to QE used for SCXPhos. However, we attempted to depreciate the influence of the MS in such variations by comparing datasets with equal coverage. Another potential origin for the differences in quantified PSRs is the phosphopeptide-enrichment buffer. We used lactic acid in SCXPhos and 2-2-2-trifluoroethanol (TFE) with potassium salts in HighPhos. Lactic acid, like other hydroxy acids, binds to TiO_2_ beads by forming a cyclic chelate more strongly than non-phosphorylated peptides, minimizing the latter’s interference in phosphopeptide enrichment [24]. However, the interaction of TFE with TiO_2_ is unclear. TFE is known to aggregate around peptides forming a matrix that is known to promote intra-peptide hydrogen bonding and creates a low dielectric environment, resulting in the increased stabilization of secondary structures of peptides rather than the denaturation of peptides [25]. It is tempting to speculate that such conditions may occur in HighPhos enrichment buffers which might interfere with the enrichment of singly phosphorylated peptides. Consistently, excluding TFE from the enrichment buffer in the updated EasyPhos workflow [9] dramatically increased identifications of singly phosphorylated peptides. In line with this interpretation, most of the quantified and IR-responsive PSRs from SCXPhos were singly phosphorylated (Figure 2C and Figure 3C), whereas PSRs from HighPhos frequently contained multiple phosphorylation events (Figure 2D and Figure 3D).

Another important issue concerns the size of the IR-responsive phosphoproteome and to what extent currently available technologies capture the entire spectrum. Firstly, our observation that the coverage of quantified phosphoproteomes increased by 150% to >30,000 total quantified phosphosites when profiled by two different methods suggests that there is method-specific bias in sampling a subset of the phosphoproteome. Secondly, our observation that the coverage increased to >45,000 phosphosites when additional IR-exposed mESC datasets from in-house projects were added and further by >400% to >82,000 phosphosites when additional mouse datasets were included suggests that the current global phosphoproteome profiling technologies are nowhere near able to capture the entire spectrum of phosphorylation events within cells, especially when considering the combinatorial possibilities. Additionally, the detection of responsive phosphosites profiled in each experiment is dependent on their coverage. The discrete overlap of 32% observed between the quantified PSRs from SCXPhos and HighPhos procedures arise mainly due to the distinct subset of phosphoproteomes being profiled by either method. This distinct subset is readily evident from their discrete composition, especially concerning the multiplicity of phosphorylation (Figure 2C,D) that could possibly arise from the chemistry of enrichment buffers used, as discussed above. This overlap is smaller than the overlap between phosphosites of two different cell lines profiled by the same method (61–68%) (Figure S2G) and is not dependent on a specific cell type or perturbation (IR exposure or EGF) (Figure S2H), exemplified by the strikingly small differences (0.3–12%) observed between various mouse tissue phosphoproteomes analyzed using similar sample preparation protocols and single-run methods (Figure S2I).

Only a minority (4%) of the IR-responsive phosphosites were identified by both methods (Figure 3B and Figure 4), most of which are single phosphorylation events and predominantly involved in the DNA damage responses. One of the main reasons for such minimal overlap in identified IR-responsive PSRs is the presence of missing quantification values for many phosphosites in either method. Bioinformatic analyses uncovered that most of the identified kinases/phosphatases, kinase–substrates and regulatory sites were disparate between these two methods. Additionally, congruent with our findings from the in-house generated dataset, method-specific phosphosite detection was also observed upon comparison of publicly available EGF-stimulated phosphoproteome datasets that were profiled using the SCXPhos or a single-run method (Figure S2H). Despite such dramatic differences in the phosphosites and kinome/phosphatome between these two methods, pathway analysis on phosphoproteins did not reveal dramatic differences. One should bear in mind that pathway analysis may not be the most appropriate tool for comparison of the biological responses of phosphosites, as they are based on the biological knowledge of proteins and genes rather than that of phosphosites.

Recently, the data-independent acquisition (DIA) technique is gaining popularity for use in phosphoproteome analysis, as it increases the phosphoproteome coverage and quantification overlap between biological replicates and samples and identifies more responsive phosphosites when compared to the most commonly used data-dependent acquisition (DDA) technique [26,27,28,29]. Among the quantification techniques, the tandem mass tags (TMT) method is gaining popularity in phosphoproteomics [22]. The TMT method has the power to productively increase phosphoproteome coverage, improve quantification overlap between replicates and improve the identification of more significantly regulated phosphosites. We speculate that combining the DIA acquisition method and the TMT labeling technique with SCXPhos and HighPhos will dramatically increase the quantification overlap (i.e., reducing missing quantification values) between biological replicates and samples, and hence will lead to the identification of more responsive phosphosites compared to the DDA and SILAC methods. However, we do not expect the overlap of the single, double, and multi-site phosphorylation events between the SCXPhos and HighPhos datasets to dramatically increase, as they seem to originate from sample preparation methods rather than the scanning modes, quantification techniques or the sequencing speed of the instrument used. More recently, parallel accumulation serial fragmentation (PASEF), a new MS scan mode [30,31,32], was established that synchronizes the novel trapped ion mobility spectrometry (TIMS) [33,34,35,36,37] form of mass spectrometry (that includes an additional separation step involving ions’ shape and size) with the MS2 precursor selection. The new aerodynamic high-field asymmetric waveform ion mobility spectrometry (FAIMS) device has also been shown to be advantageous for phosphoproteomics experiments by improving the coverage and quantifications of phosphosites [38,39,40,41,42,43,44,45]. However, large-scale studies are required to unveil the impact of the various above-described advancements in instrumentation, scanning modes and quantification techniques when combined with SCXPhos and HighPhos sample preparation methods for phosphoproteomics. Our experience with measuring the HighPhos sample using different machines in the DDA setting suggests that the composition of the uncovered phosphoproteome is not expected to be affected by these aspects. Nevertheless, this assumption should be verified using the various above-described novel techniques.

### Limitations of the Study

The comparative phosphoproteome analysis presented in this study involves using a single quantification technique (SILAC), a single perturbation (ionizing radiation), a single data acquisition method (DDA), two enzymes (Trypsin and Lys-C) and a single enrichment bead (TiO_2_). Hence, the influence of each of these components on the quantification of more phosphosites or the identification of additional biology is unclear. Future studies focusing on expanding on these aspects should clarify this point. Moreover, the ‘match between runs’ (MBR) algorithm implemented in MaxQuant inherently does not include an FDR control, and hence the results obtained from this step may contain false positives, even for a smaller number of raw files, which is a downside of this computational step. The implementation of a statistical control step by developers of MaxQuant software in future versions would allow correction for false positives. We ask that investigators use this MBR step with caution and verify results obtained from MBR using other techniques, such as spiking-in labeled phosphopeptides.

CPA: While our complex setup involving a Windows virtual machine running inside a Linux server accessed via cloud computing is feasible, we suggest that investigators consider the moderate outcome achieved from this endeavor before embarking on such approaches and recommend running it on native Windows servers directly for high-speed processing. The reported 35 days of computational time using MaxQuant is mainly due to the complexity of the MS spectra imparted by triple SILAC labeling, which dramatically increases the MaxQuant run times compared to label-free samples, especially in combination with the phospho(STY) modification search. However, for proteomics labs that wish to perform label-free quantification on numerous measurements, this task should be hassle-free due to reduced spectral complexity (thus increasing the speed of computation), and hence it is realistic when performed on native Windows servers.

## 5. Conclusions

These observations suggest that the role of sample preparation methods in uncovering the full spectrum of responsive phosphosites in large-scale phosphoproteomics studies is grossly underestimated. Our data demonstrate that the inclusion of multiple biological replicates and the combination of multiple sample preparation methods are advantageous to uncover a broader spectrum of phosphorylation signaling biology.

## Data Availability

Data are available via ProteomeXchange with identifier PXD025376.

## Supplementary Materials

The following are available online at https://www.mdpi.com/article/10.3390/cells10123407/s1, Figure S1: experimental setup for methods comparison, Figure S2: cumulative phosphoproteome analysis, Figure S3: pathways enriched from PSRs at 0.5 h after IR exposure, Figure S4: pathways enriched from PSRs at 4 h after IR exposure, Figure S5: phosphorylation dynamics of key signal integrators, Figure S6: heatmap of kinome/phosphatome, Figure S7: pathways enriched from PSRs after LD exposure, Figure S8: pathways enriched from PSRs after HD exposure. Table S1: Quantified phosphosites before CPA, Table S2: Quantified phosphosites after CPA, Table S3: IR-responsive phosphosites (before CPA).
