# Supplementary material for: Phosphoproteomics Sample Preparation Impacts Biological Interpretation of Phosphorylation Signaling Outcomes
Source: Cells. 2021 Dec 3;10(12):3407. doi: 10.3390/cells10123407 (PMC8699897; doi:10.3390/cells10123407)
Supplement: Supplementary file 1 [file cells-10-03407-s001.zip › cells-1460896-supplementary/cells-1460896-supplementary-figures.pdf]

Figure S1

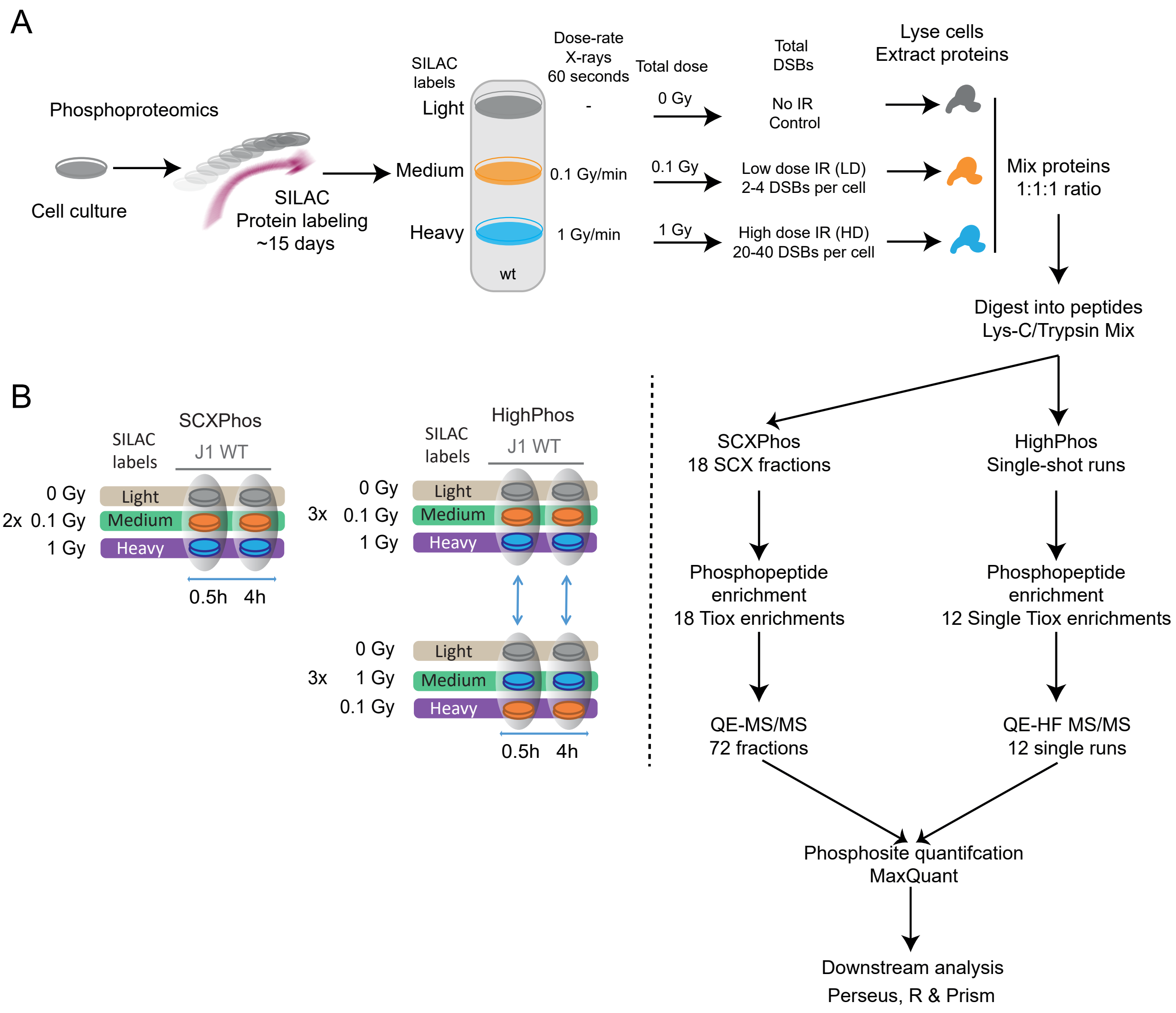
A
Phosphoproteomics
Cell culture
SILAC
Protein labeling
~15 days
SILAC
labels
Light
Medium
Heavy
wt
Dose-rate
X-rays
60 seconds
-
0.1 Gy/min
1 Gy/min
Total dose
0 Gy
0.1 Gy
1 Gy
Total
DSBs
No IR
Control
Low dose IR (LD)
2-4 DSBs per cell
High dose IR (HD)
20-40 DSBs per cell
Lyse cells
Extract proteins
Mix proteins
1:1:1 ratio
Digest into peptides
Lys-C/Trypsin Mix
B
SCXPhos
SILAC
labels
J1 WT
0 Gy
2x 0.1 Gy
1 Gy
Light
Medium
Heavy
0.5h 4h
HighPhos
SILAC
labels
J1 WT
0 Gy
3x 0.1 Gy
1 Gy
Light
Medium
Heavy
0 Gy
3x 1 Gy
0.1 Gy
Light
Medium
Heavy
0.5h 4h
SCXPhos
18 SCX fractions
HighPhos
Single-shot runs
Phosphopeptide
enrichment
18 Tiox enrichments
Phosphopeptide
enrichment
12 Single Tiox enrichments
QE-MS/MS
72 fractions
QE-HF MS/MS
12 single runs
Phosphosite quantifcation
MaxQuant
Downstream analysis
Perseus, R & Prism

Figure S1: Experimental setup for methods comparison

(A) General workflow of the phosphoproteomics experiment. Mouse ESCs were cultured in SILAC media for over 7 passages for a period of 15 days to complete protein labelling. SILAC-labelled mESCs were then exposed to IR and lysates were prepared and processed. Subsequently, phosphopeptides were enriched, sequenced by a mass spectrometer and data were analysed. J1 mESCs were exposed to X-rays for 60 seconds at a dose-rate of 0.1Gy/min for low dose IR (LD) (2-4 DSBs per cell) and 1 Gy/min for high dose IR (HD) (20-40 DSBs per cell). Control samples were mock-treated. Cells were SILAC labelled with either "Light", "Medium" or "Heavy" labels and were mock-, LD- or HD- treated respectively. Lysates were prepared from each sample, SILAC-labelled protein lysates were multiplexed in equal ratio (1:1:1) based on protein quantity, digested into tryptic peptides and processed via two different phosphoproteomics workflows. For SCXPhos experiments, samples were fractionated (eighteen fractions) using strong cation exchange (SCX) chromatography followed by phosphopeptide enrichment using TiO_2 chromatography. In total, 72 fractions were analysed by Q-Exactive MS. For HighPhos experiments, samples are directly processed and enriched for phosphopeptides without fractionation and sequenced by Q-Exactive HF MS. In total, 12 single-run samples were analysed. **(B)** For SCXPhos, both replicates of J1 mESCs were SILAC-labelled with either "Light", "Medium" or "Heavy" labels and were mock-, LD- or HD- treated respectively. For HighPhos, we performed a label swap experiments. In three of the six replicates, "Medium" and "Heavy" SILAC-labelled mESCs were exposed to LD and HD, respectively. In the remaining three replicates, "Heavy" and "Medium" SILAC-labelled mESCs were exposed to LD and HD, respectively. In all six replicates "Light" SILAC-labelled cells were mock-treated. For both methods, samples were collected at 0.5h and 4h after IR exposure.

Figure S2

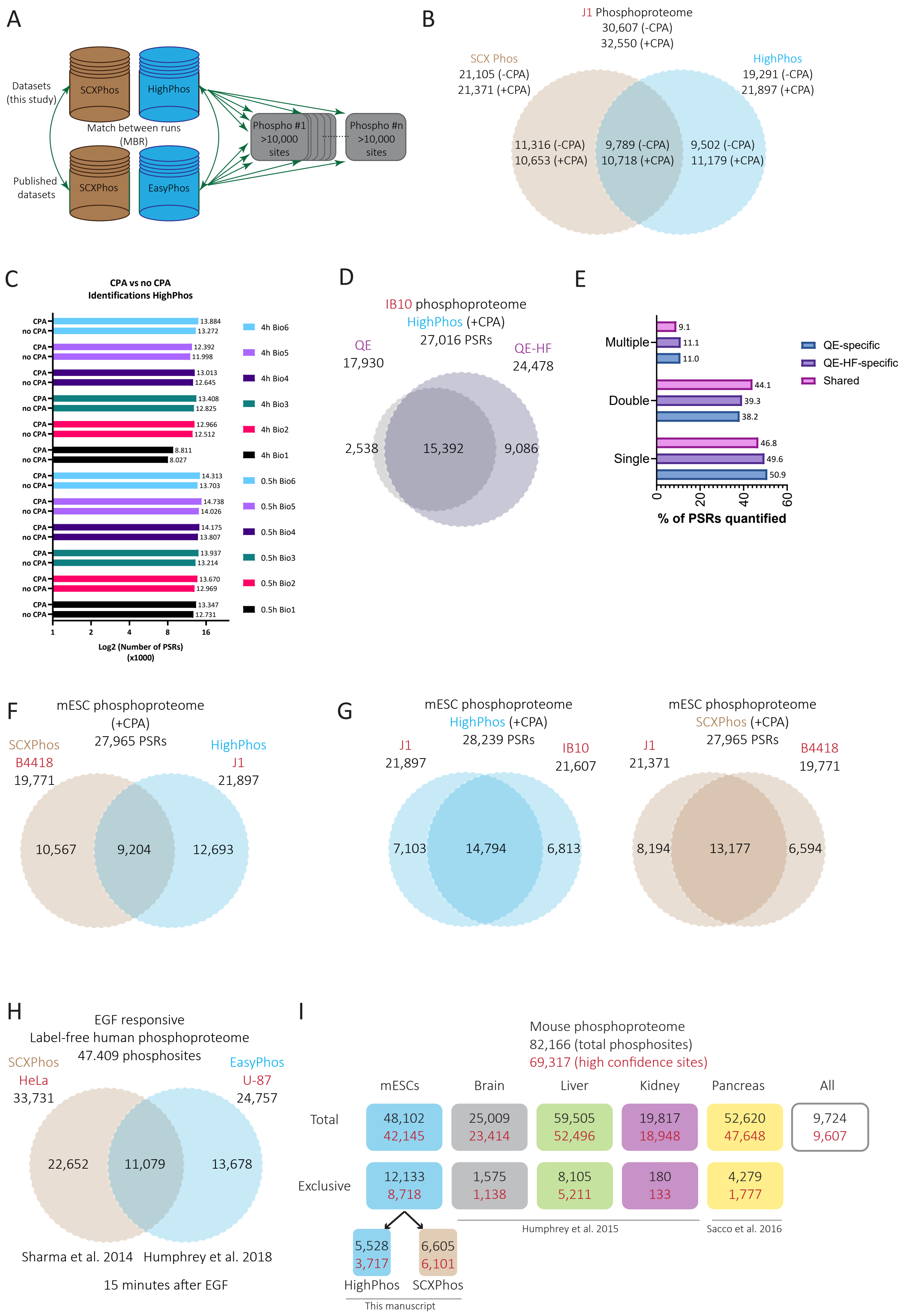
A
Datasets (this study)
SCXPhos
HighPhos
Match between runs (MBR)
Published datasets
SCXPhos
EasyPhos
Phospho #1 >10,000 sites
Phospho #n >10,000 sites
B
J1 Phosphoproteome
30,607 (-CPA)
32,550 (+CPA)
SCX Phos
21,105 (-CPA)
21,371 (+CPA)
HighPhos
19,291 (-CPA)
21,897 (+CPA)
11,316 (-CPA)
10,653 (+CPA)
9,789 (-CPA)
10,718 (+CPA)
9,502 (-CPA)
11,179 (+CPA)
C
CPA vs no CPA
Identifications HighPhos
CPA 13.884 no CPA 13.272 4h Bio6
CPA 12.392 no CPA 11.998 4h Bio5
CPA 13.013 no CPA 12.645 4h Bio4
CPA 13.408 no CPA 12.825 4h Bio3
CPA 12.966 no CPA 12.512 4h Bio2
CPA 8.811 no CPA 8.027 4h Bio1
CPA 14.313 no CPA 13.703 0.5h Bio6
CPA 14.738 no CPA 14.026 0.5h Bio5
CPA 14.175 no CPA 13.807 0.5h Bio4
CPA 13.937 no CPA 13.214 0.5h Bio3
CPA 13.670 no CPA 12.969 0.5h Bio2
CPA 13.347 no CPA 12.731 0.5h Bio1
1 2 4 8 16
Log2 (Number of PSRs) (x1000)
D
IB10 phosphoproteome
HighPhos (+CPA)
27,016 PSRs
QE
17,930
QE-HF
24,478
2,538
15,392
9,086
E
Multiple
9.1
11.1
11.0
Double
44.1
39.3
38.2
Single
46.8
49.6
50.9
0 20 40 60
% of PSRs quantified
QE-specific
QE-HF-specific
Shared
F
mESC phosphoproteome (+CPA)
27,965 PSRs
SCXPhos
B4418
19,771
HighPhos
J1
21,897
10,567
9,204
12,693
G
mESC phosphoproteome
HighPhos (+CPA)
28,239 PSRs
J1
21,897
IB10
21,607
7,103
14,794
6,813
mESC phosphoproteome
SCXPhos (+CPA)
27,965 PSRs
J1
21,371
B4418
19,771
8,194
13,177
6,594
H
EGF responsive
Label-free human phosphoproteome
47.409 phosphosites
SCXPhos
HeLa
33,731
EasyPhos
U-87
24,757
22,652
11,079
13,678
Sharma et al. 2014
Humphrey et al. 2018
15 minutes after EGF
I
Mouse phosphoproteome
82,166 (total phosphosites)
69,317 (high confidence sites)
mESCs
Brain
Liver
Kidney
Pancreas
All
Total
48,102 42,145
25,009 23,414
59,505 52,496
19,817 18,948
52,620 47,648
9,724 9,607
Exclusive
12,133 8,718
1,575 1,138
8,105 5,211
180 133
4,279 1,777
5,528 3,717
6,605 6,101
HighPhos
SCXPhos
This manuscript
Humphrey et al. 2015
Sacco et al. 2016

Figure S2: Cumulative phosphoproteome analysis

(A) Schematic diagram depicting the cumulative phosphoproteome analysis (CPA) strategy used to analyse the publicly available mouse EasyPhos datasets together with our raw MS data. **(B)** Venn diagram depicting the overlap of number of PSRs quantified in SCXPhos (brown circle) and HighPhos (blue circle). The number of PSRs quantified before (-CPA) and after CPA (+CPA) is indicated for each dataset. **(C)** Bar chart showing number of phosphosite identifications before and after CPA for the HighPhos dataset. **(D)** Venn diagram showing the overlap of number of PSRs quantified by Q Exactive (QE; grey circle) and Q Exactive-HF (QE-HF; purple circle) mass spectrometers **(E)** Bar chart displaying the percentage of single, double or multiple phosphorylation events among QE-specific (blue bars), QE-HF-specific (purple bars) and shared between QE and QE-HF (pink bars). **(F)** Venn diagram depicting the overlap of number of PSRs quantified in B4418 SCXPhos (brown circle) and J1 HighPhos (blue circle). **(G)** Venn diagram depicting the overlap of number of PSRs quantified in J1 and IB10 mESCs using the HighPhos (blue circle) method and J1 and B4418 mESCs using the SCXPhos (brown circle) method. **(H)** Venn diagram depicting the overlap of phosphosites quantified in HeLa cell line using the SCXPhos method (brown circle) and U-87 cell line using the single-run method (blue circle) 15 minutes after EGF stimulation. **(I)** Comparison of quantified phosphosites in mouse ESCs, liver, brain, kidney and pancreas. For each dataset, top (black) and bottom (red) numbers represent the total and high-confidence phosphosites. Numbers for mESCs are combined from both HighPhos and SCXPhos datasets. Numbers for other tissues are all from single-run EasyPhos analyses.

Figure S3

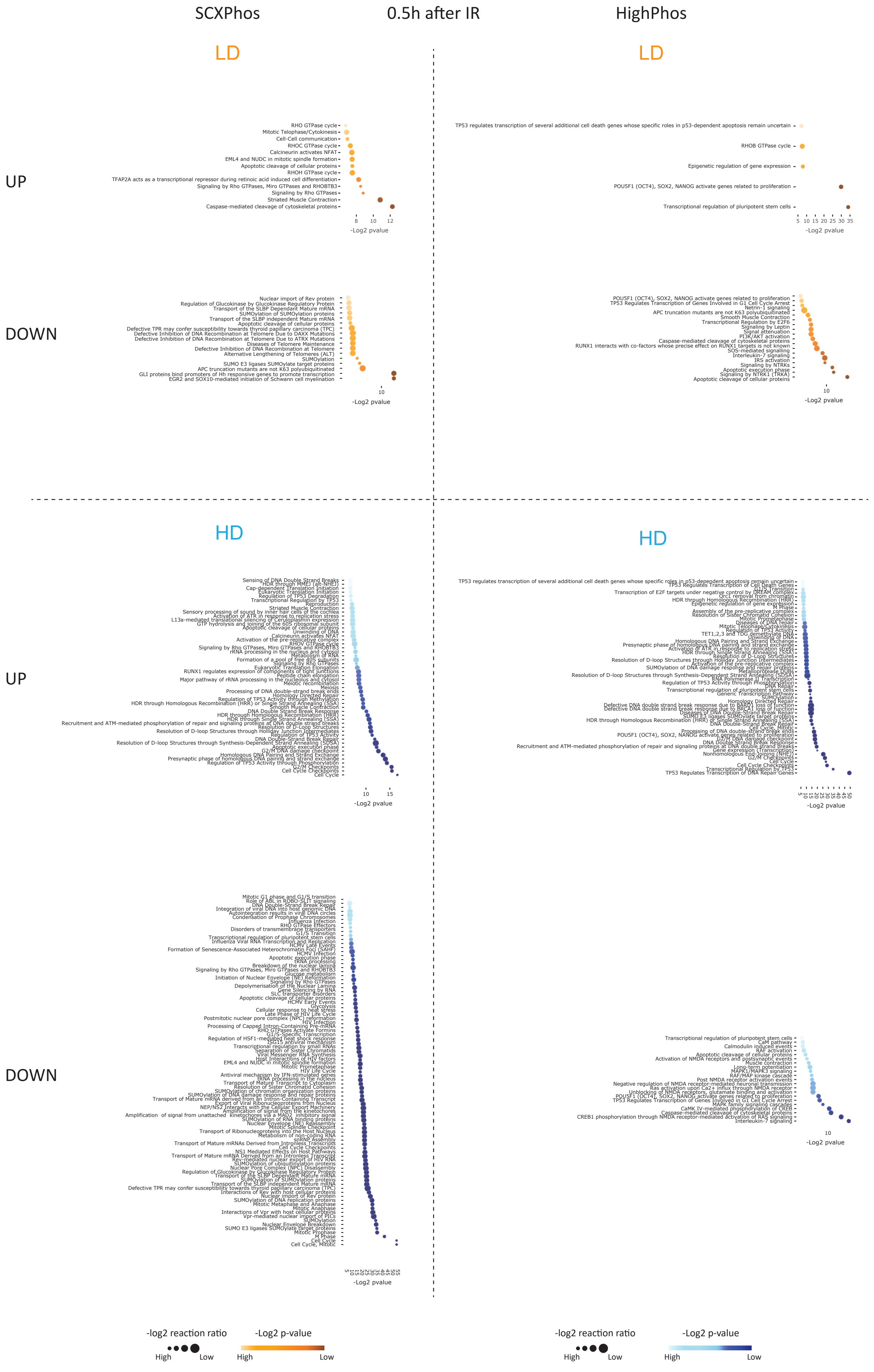
SCXPhos
0.5h after IR
HighPhos
LD
HD
UP
DOWN
-Log2 pvalue
-log2 reaction ratio
High
Low
-Log2 p-value
High
Low

Figure S3: Pathways enriched from PSRs at 0.5h after IR exposure

Gene ontology (GO) analysis of phosphoproteins representing up and down-regulated PSRs at 0.5h after LD- (top panel) and HD- (bottom panel) exposed cells from SCXPhos (left panel and HighPhos (right panel) experiment using reactome pathways.

Figure S4

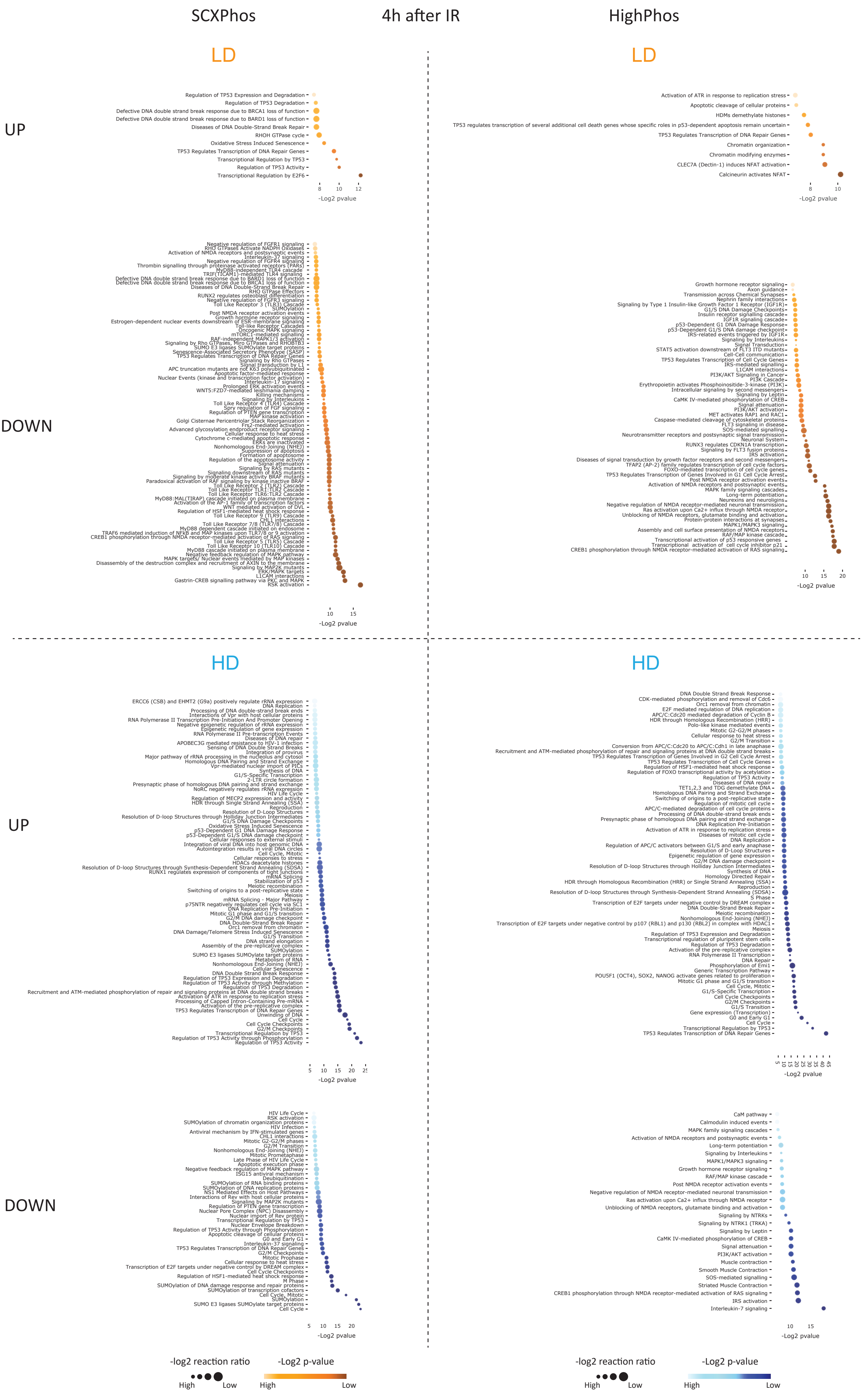
SCXPhos
4h after IR
HighPhos
LD
UP
DOWN
HD
-Log2 pvalue
-log2 reaction ratio
-Log2 p-value
High
Low

Figure S4: Pathways enriched from PSRs at 4h after IR exposure

Gene ontology (GO) analysis of phosphoproteins representing up- and down-regulated PSRs at 4h after LD- (top panel) and HD- (bottom panel) exposed cells from SCXPhos (left panel and HighPhos (right panel) experiment using reactome pathways.

Figure S5

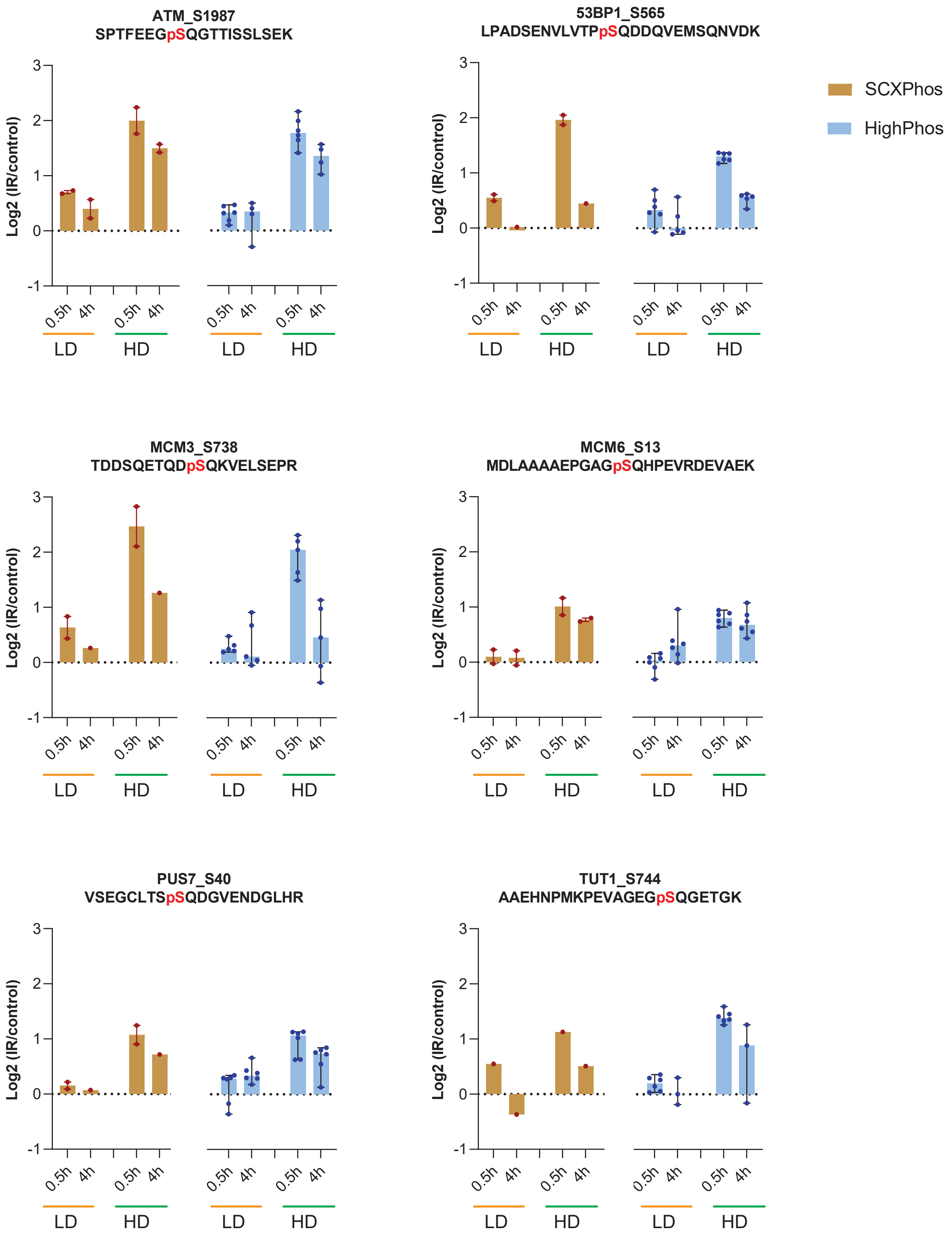
ATM_S1987
SPTFEEGpSQGTTISSLSEK
53BP1_S565
LPADSENVLVTPpSQDDQVEMSQNVDK
MCM3_S738
TDDSQETQDpSQKVELSEPR
MCM6_S13
MDLAAAAEPGAGpSQHPEVRDEVAEK
PUS7_S40
VSEGCLTSpSQDGVENDGLHR
TUT1_S744
AAEHNPMKPEVAGEGpSQGETGK
SCXPhos
HighPhos
Log2 (IR/control)
0.5h
4h
LD
HD

Figure S5: Phosphorylation dynamics of key signal integrators

Bar charts showing phosphorylation levels of ATM pS1987, 53BP1 pS565, MCM3 pS738, MCM6 pS13, PUS7 pS40 and TUT1 pS744. Brown and blue bars represent data from SCXPhos and HighPhos respectively. Dots represent quantifications of up to six replicates. Bars represent median values with 95% confident interval (CI) indicated as error bars.

Figure S6

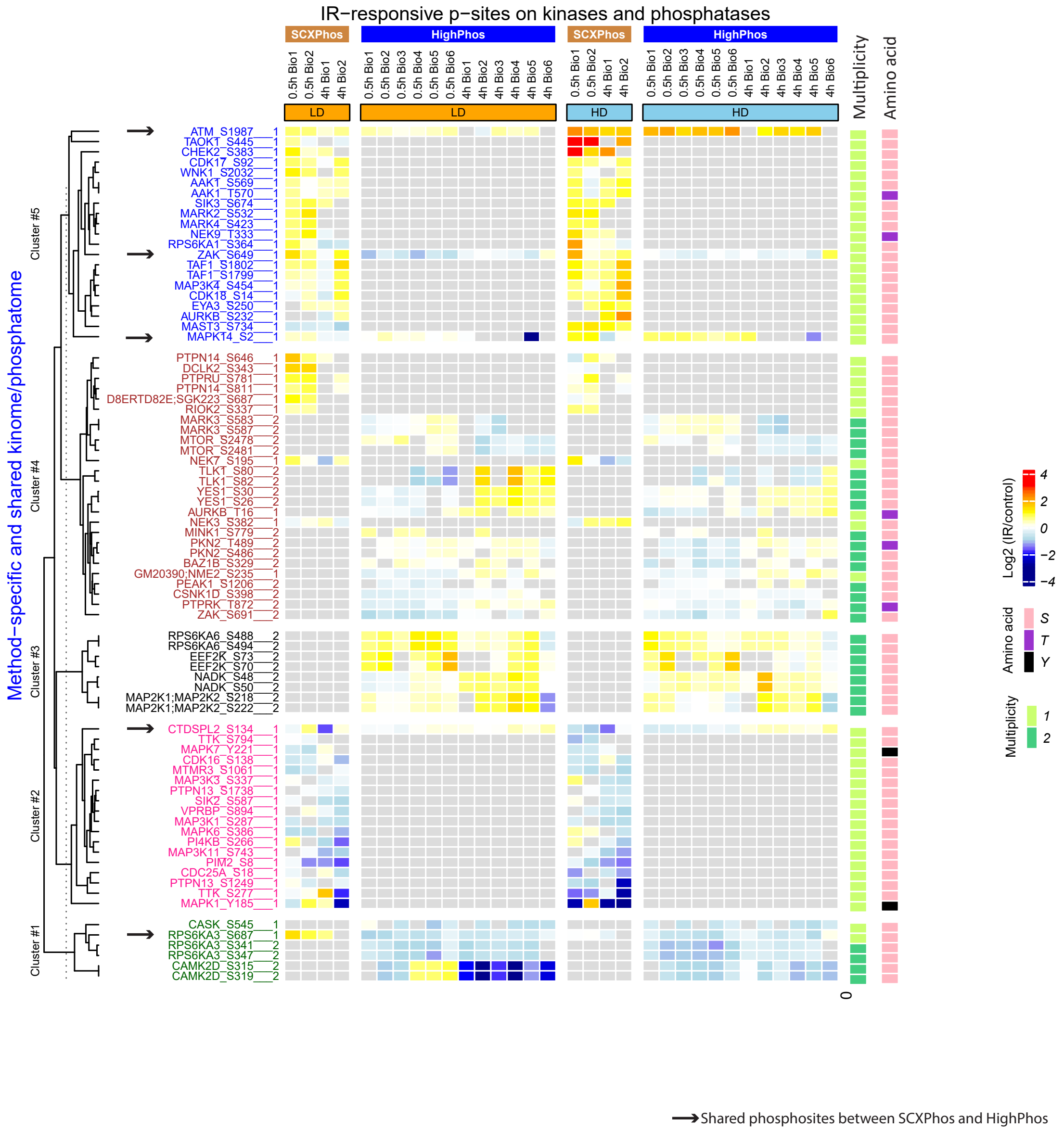

IR−responsive p−sites on kinases and phosphatases
SCXPhos
HighPhos
SCXPhos
HighPhos
0.5h Bio1
0.5h Bio2
4h Bio1
4h Bio2
0.5h Bio3
0.5h Bio4
0.5h Bio5
0.5h Bio6
4h Bio3
4h Bio4
4h Bio5
4h Bio6
LD
HD
Multiplicity
Amino acid
Method−specific and shared kinome/phosphatome
Cluster #5
Cluster #4
Cluster #3
Cluster #2
Cluster #1
ATM_S1987___1
TAOK1_S445___1
CHEK2_S383___1
CDK17_S92___1
WNK1_S2032___1
AAK1_S569___1
AAK1_T570___1
SIK3_S674___1
MARK2_S532___1
MARK4_S423___1
NEK9_T333___1
RPS6KA1_S364___1
ZAK_S649___1
TAF1_S1802___1
TAF1_S1799___1
MAP3K4_S454___1
CDK18_S14___1
EYA3_S250___1
AURKB_S232___1
MAST3_S734___1
MAPK14_S2___1
PTPN14_S646___1
DCLK2_S343___1
PTPRU_S781___1
PTPN14_S811___1
D8ERTD82E;SGK223_S687___1
RIOK2_S337___1
MARK3_S583___2
MARK3_S587___2
MTOR_S2478___2
MTOR_S2481___2
NEK7_S195___1
TLK1_S80___2
TLK1_S82___2
YES1_S30___2
YES1_S26___2
AURKB_T16___1
NEK3_S382___1
MINK1_S779___2
PKN2_T489___2
PKN2_S486___2
BAZ1B_S329___2
GM20390;NME2_S235___1
PEAK1_S1206___2
CSNK1D_S398___2
PTPRK_T872___2
ZAK_S691___2
RPS6KA6_S488___2
RPS6KA6_S494___2
EEF2K_S73___2
EEF2K_S70___2
NADK_S48___2
NADK_S50___2
MAP2K1;MAP2K2_S218___2
MAP2K1;MAP2K2_S222___2
CTDSPL2_S134___1
TTK_S794___1
MAPK7_Y221___1
CDK16_S138___1
MTMR3_S1061___1
MAP3K3_S337___1
PTPN13_S1738___1
SIK2_S587___1
VPRBP_S894___1
MAP3K1_S287___1
MAPK6_S386___1
PI4KB_S266___1
MAP3K11_S743___1
PIM2_S8___1
CDC25A_S18___1
PTPN13_S1249___1
TTK_S277___1
MAPK1_Y185___1
CASK_S545___1
RPS6KA3_S687___1
RPS6KA3_S341___2
RPS6KA3_S347___2
CAMK2D_S315___2
CAMK2D_S319___2
0
Log2 (IR/control)
4
2
0
−2
−4
Amino acid
S
T
Y
Multiplicity
1
2
Shared phosphosites between SCXPhos and HighPhos

Figure S6: Heatmap of kinome/phosphatome

Heatmap of the IR-responsive PSRs of kinases and phosphatases from SCXPhos and HighPhos experiments after LD (left panel) and HD (right panel) exposures. Hierarchical clustering was performed to order phosphosites. Grey blocks indicate NQ (no quantifications). Hierarchical clustering was performed to order phosphosites. Phosphosites labels should be read as follows: Gene_phosphorylated amino acid phosphorylated residue___multiplicity. Multiplicity represents the number of phosphorylation events observed. For each phosphosite, the phosphorylated amino acid, and the number of phosphorylation events observed are annotated on the right side of the heatmap.

Figure S7

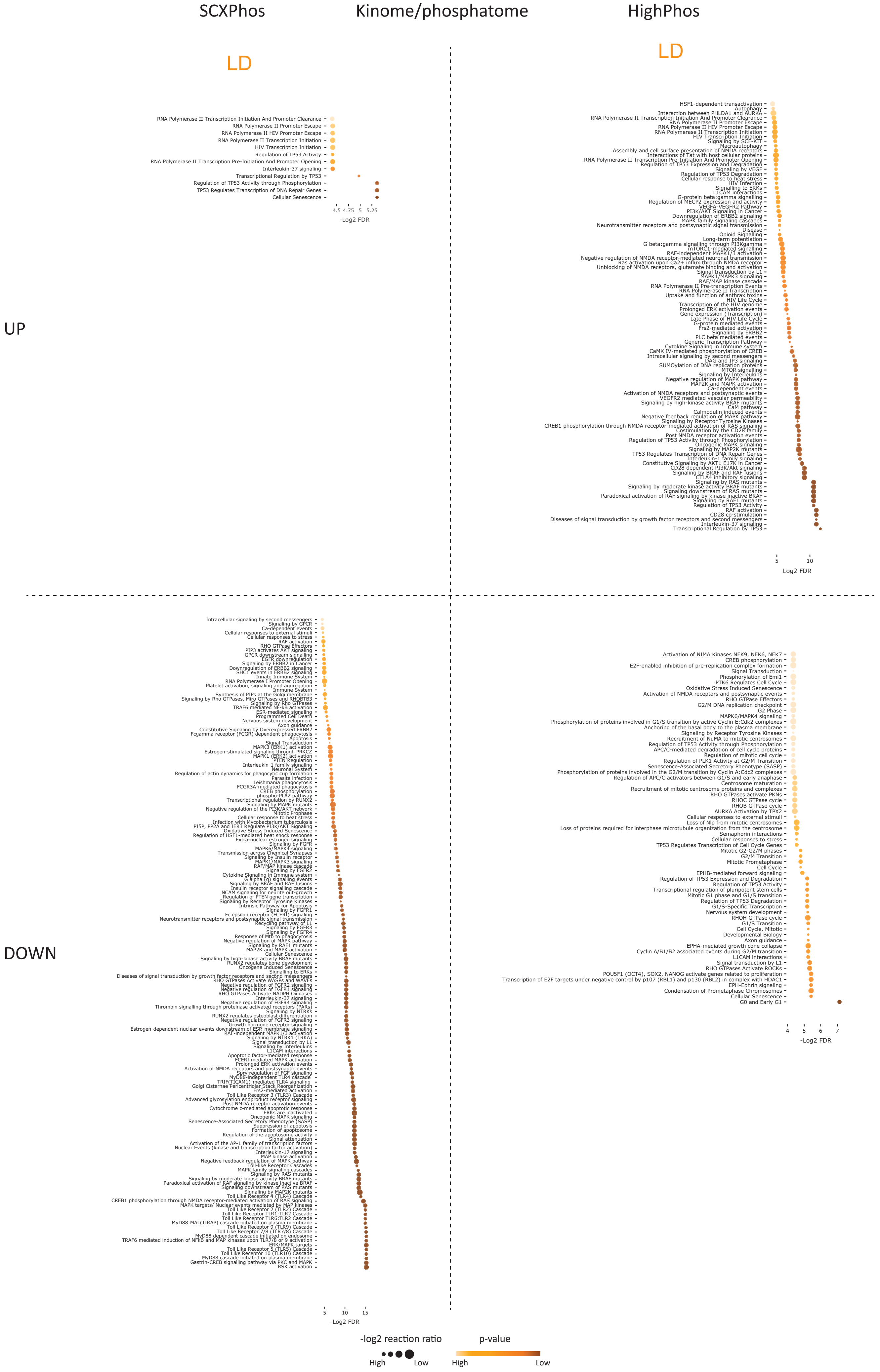
SCXPhos
Kinome/phosphatome
HighPhos
LD
LD
UP
DOWN
-Log2 FDR
-log2 reaction ratio
High
Low
p-value
High
Low

Figure S7: Pathways enriched from PSRs after LD exposure

Gene ontology (GO) analysis of phosphoproteins representing up-regulated (top panel) and down-regulated (bottom panel) PSRs from SCXPhos (left panel and HighPhos (right panel) experiment after LD exposure using reactome pathways.

Figure S8

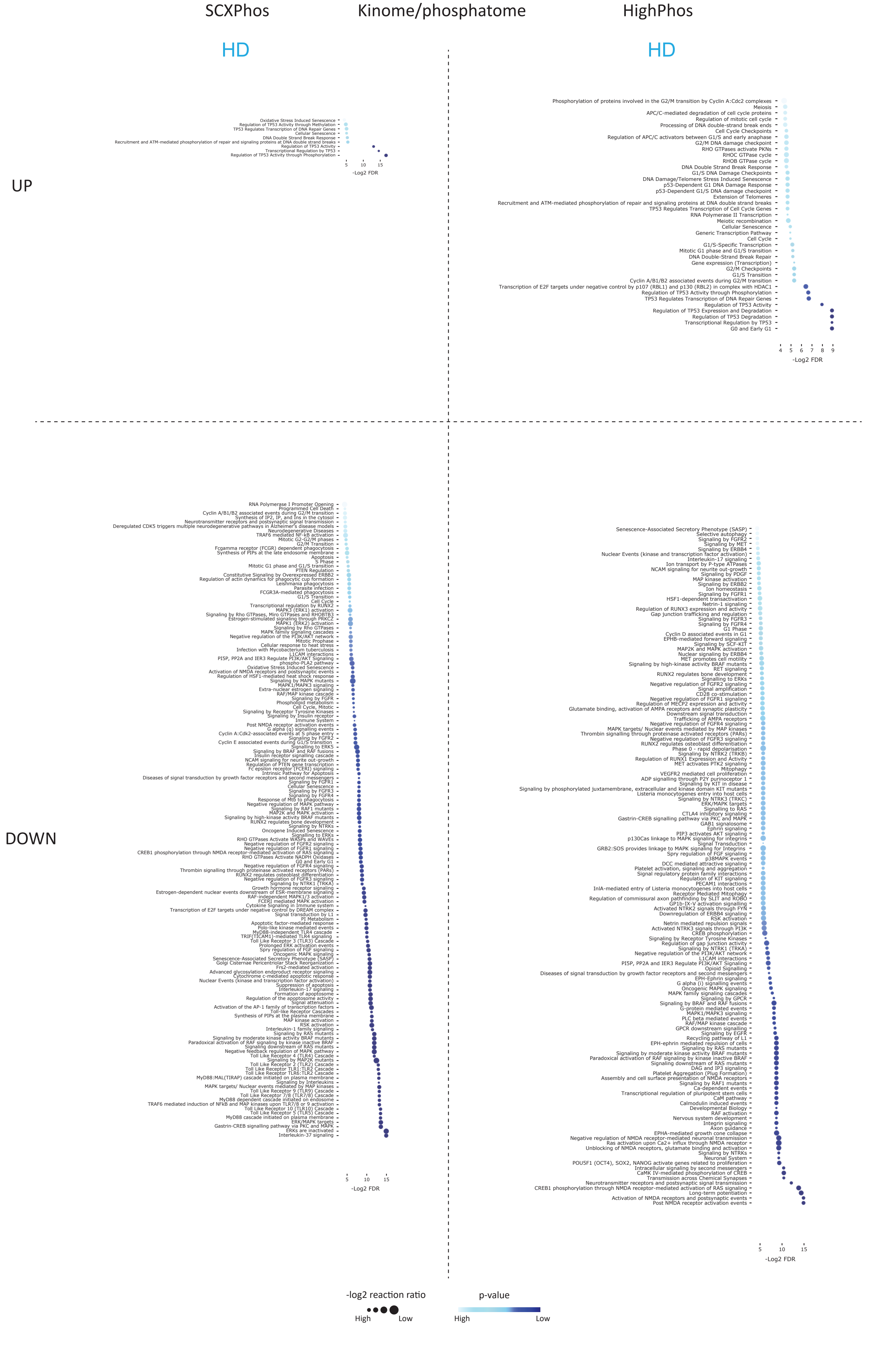
SCXPhos
Kinome/phosphatome
HighPhos
HD
HD
UP
DOWN
Oxidative Stress Induced Senescence
Regulation of TP53 Activity through Methylation
TP53 Regulates Transcription of DNA Repair Genes
Cellular Senescence
DNA Double Strand Break Response
Recruitment and ATM-mediated phosphorylation of repair and signaling proteins at DNA double strand breaks
Regulation of TP53 Activity
Transcriptional Regulation by TP53
Regulation of TP53 Activity through Phosphorylation
-Log2 FDR
Phosphorylation of proteins involved in the G2/M transition by Cyclin A:Cdc2 complexes
Meiosis
APC/C-mediated degradation of cell cycle proteins
Regulation of mitotic cell cycle
Processing of DNA double-strand break ends
Cell Cycle Checkpoints
Regulation of APC/C activators between G1/S and early anaphase
G2/M DNA damage checkpoint
RHO GTPases activate PKNs
RHOC GTPase cycle
RHOB GTPase cycle
DNA Double Strand Break Response
G1/S DNA Damage Checkpoints
DNA Damage/Telomere Stress Induced Senescence
p53-Dependent G1 DNA Damage Response
p53-Dependent G1/S DNA damage checkpoint
Extension of Telomeres
Recruitment and ATM-mediated phosphorylation of repair and signaling proteins at DNA double strand breaks
TP53 Regulates Transcription of Cell Cycle Genes
RNA Polymerase II Transcription
Meiotic recombination
Cellular Senescence
Generic Transcription Pathway
Cell Cycle
G1/S-Specific Transcription
Mitotic G1 phase and G1/S transition
DNA Double-Strand Break Repair
Gene expression (Transcription)
G2/M Checkpoints
G1/S Transition
Cyclin A/B1/B2 associated events during G2/M transition
Transcription of E2F targets under negative control by p107 (RBL1) and p130 (RBL2) in complex with HDAC1
Regulation of TP53 Activity through Phosphorylation
TP53 Regulates Transcription of DNA Repair Genes
Regulation of TP53 Activity
Regulation of TP53 Expression and Degradation
Regulation of TP53 Degradation
Transcriptional Regulation by TP53
G0 and Early G1
-Log2 FDR
-log2 reaction ratio
High
Low
p-value
High
Low

Figure S8: Pathways enriched from PSRs after HD exposure

Gene ontology (GO) analysis of phosphoproteins representing up-regulated (top panel) and down-regulated (bottom panel) PSRs from SCXPhos (left panel and HighPhos (right panel) experiment after HD exposure using reactome pathways.
